# A Viral Suppressor Modulates the Plant Immune Response Early in Infection by Regulating MicroRNA Activity

**DOI:** 10.1128/mBio.00419-18

**Published:** 2018-04-24

**Authors:** Robert Pertermann, Selvaraj Tamilarasan, Torsten Gursinsky, Giorgio Gambino, Jana Schuck, Claus Weinholdt, Hauke Lilie, Ivo Grosse, Ralph Peter Golbik, Vitantonio Pantaleo, Sven-Erik Behrens

**Affiliations:** a Institute of Biochemistry and Biotechnology, Martin Luther University Halle-Wittenberg, Halle/Saale, Germany; b Institute for Sustainable Plant Protection-Consiglio Nazionale delle Ricerche, Turin, Italy; c Institute of Informatics, Martin Luther University Halle-Wittenberg, Halle/Saale, Germany; d Institute for Sustainable Plant Protection-Consiglio Nazionale delle Ricerche, Research Unit of Bari, Bari, Italy; Columbia University College of Physicians & Surgeons

**Keywords:** RISC, RNA interference, RNA replication, RNA silencing, RNA-protein interactions, VSR, antiviral, immune evasion, miRNA, plant viruses, plus-strand RNA virus, siRNA

## Abstract

Many viral suppressors (VSRs) counteract antiviral RNA silencing, a central component of the plant’s immune response by sequestration of virus-derived antiviral small interfering RNAs (siRNAs). Here, we addressed how VSRs affect the activities of cellular microRNAs (miRNAs) during a viral infection by characterizing the interactions of two unrelated VSRs, the *Tombusvirus* p19 and the *Cucumovirus* 2b, with miRNA 162 (miR162), miR168, and miR403. These miRNAs regulate the expression of the important silencing factors Dicer-like protein 1 (DCL1) and Argonaute proteins 1 and 2 (AGO1 and AGO2), respectively. Interestingly, while the two VSRs showed similar binding profiles, the miRNAs were bound with significantly different affinities, for example, with the affinity of miR162 greatly exceeding that of miR168. *In vitro* silencing experiments revealed that p19 and 2b affect miRNA-mediated silencing of the *DCL1*, *AGO1*, and *AGO2* mRNAs in strict accordance with the VSR’s miRNA-binding profiles. In *Tombusvirus*-infected plants, the miRNA-binding behavior of p19 closely corresponded to that *in vitro*. Most importantly, in contrast to controls with a Δp19 virus, infections with wild-type (wt) virus led to changes of the levels of the miRNA-targeted mRNAs, and these changes correlated with the miRNA-binding preferences of p19. This was observed exclusively in the early stage of infection when viral genomes are proposed to be susceptible to silencing and viral siRNA (vsiRNA) concentrations are low. Accordingly, our study suggests that differential binding of miRNAs by VSRs is a widespread viral mechanism to coordinately modulate cellular gene expression and the antiviral immune response during infection initiation.

## IMPORTANCE

Plant viruses manipulate their hosts in various ways. Viral suppressor proteins (VSRs) interfere with the plant’s immune response by sequestering small, antivirally acting vsiRNAs, which are processed from viral RNAs during the plant’s RNA-silencing response. Here, we examined the effects of VSRs on cellular microRNAs (miRNAs), which show a high degree of similarity with vsiRNAs. Binding experiments with two unrelated VSRs and three important regulatory miRNAs revealed that the proteins exhibit similar miRNA-binding profiles but bind different miRNAs at considerably different affinities. Most interestingly, experiments in plants showed that in the early infection phase, the *Tombusvirus* VSR p19 modulates the activity of these miRNAs on their target mRNAs very differently and that this differential regulation strictly correlates with the binding affinities of p19 for the respective miRNAs. Our data suggest that VSRs may specifically control plant gene expression and the early immune response by differential sequestration of miRNAs.

## INTRODUCTION

RNA silencing regulates gene expression on the transcriptional or posttranscriptional levels and decisively affects multiple biological processes in eukaryotic cells. In plants, RNA silencing is a major element of the immune response against pathogens, including viruses (1, 2). During infections with plus-strand RNA viruses, which represent the majority of plant-infecting viruses, antiviral RNA silencing is induced by double-stranded RNAs (dsRNAs) of viral origin. These may be structured regions of the viral genomes, dsRNA viral replication intermediates, or dsRNAs that are generated from viral RNA templates by host RNA-dependent RNA polymerases (RDRs) (3, 4). Viral dsRNAs are mainly detected and processed by the Dicer-like proteins DCL4 and DCL2, which generate 21- to 22-nucleotide (nt) viral small interfering RNA (vsiRNA) duplexes (5, 6). In the course of an infection, vsiRNAs gradually accumulate and may be further amplified by RDRs (7–9). The spread of primary and secondary (RDR-generated) vsiRNAs then may lead to a reduction in virus titer and to the induction of local and systemic plant immunity (10, 11).

The major effectors of RNA silencing are Argonaute (AGO) nucleases (12) that are the active components of as-yet incompletely characterized RNA-induced silencing complexes (RISC) (13–15). Of the 10 AGO proteins that have been identified in Arabidopsis thaliana, AGO1, -2, -3, -5, -7, and -10 were shown to have antiviral RNA-silencing activity (16). Following the incorporation of a vsiRNA into an AGO protein, one strand, the passenger strand, is removed (17, 18), while the remaining guide strand (g-strand) directs the RISC to the cognate viral RNA. The best-characterized antivirally acting AGO1 and AGO2 proteins contribute to the removal of viral RNA or subviral entities by “slicing,” i.e., endonucleolytic cleavage, of the target RNA in a vsiRNA-directed, sequence-specific manner (19).

Most viruses encode one or more *v*iral *s*uppressors of *R*NA silencing (VSRs), which may block antiviral RNA silencing at different stages (20). One of the best-characterized VSRs is the *Tombusvirus* p19 protein, which was shown to act in siRNA sequestration. It binds siRNA duplexes with high affinity, and binding is dependent on size but not on sequence of the RNA molecule (21–23) (see also below). p19-dependent sequestration of siRNAs prevents RISC assembly (24–26) and interferes with the systemic spread of silencing. Another example is the *Cucumovirus* 2b protein, which also suppresses RNA silencing through the binding of dsRNAs of various sizes (27, 28). As with most siRNA-binding VSRs of different virus species, the primary protein structures of p19 and 2b are entirely unrelated (20).

RNA silencing also regulates plant gene expression on the posttranscriptional level by RISC containing 21- to 22-nucleotide, noncoding microRNAs (miRNAs) targeting cellular mRNAs. miRNA duplexes are processed from cellular pri-miRNA transcripts involving the Dicer-like protein DCL1 (29). Following the sorting into AGO1/RISC, the passenger strand (also known as the “star strand” [* strand]) is removed. The miRNA-programmed RISC activity involves slicing; alternatively, it may sterically block the recruitment or movement of ribosomes during translation (30).

An important topic of current research concerns the autoregulation of RNA silencing in plants. In dicots, three miRNAs were indicated to have central roles in this context. miRNA 162 (miR162) targets the mRNA of DCL1 (31), miR168 targets the mRNA of AGO1 (32), and miR403 targets the mRNAs of the related AGO2 and AGO3 (33). RISC containing miR168 or miR403 may accordingly cleave or translationally inhibit the *AGO1* or *AGO2* mRNAs in a feedback-like manner. Best investigated in this respect is the homeostasis of AGO1 (34), which is considerably affected during viral infections. Thus, p19 as well as other VSRs was shown to promote an induction of miR168 accumulation by a yet-unknown mechanism and, as a result, a posttranscriptional repression of the synthesis of the antiviral AGO1 protein (35, 36).

The observation that VSRs induce the expression of AGO-regulating miRNAs prompted the question whether these proteins also interfere with miRNA activity, e.g., by sequestering miRNAs in a similar way as siRNAs. Several reports suggested that VSRs may indeed interfere with miRNA pathways (37–41). Experimental data obtained with a transgenic, p19-expressing A. thaliana line showed that p19 is capable of recruiting miRNAs and that this may prevent RISC loading (42). Furthermore, p19 was shown to bind miRNA duplexes and to stabilize the otherwise rapidly degraded miRNA star strands (43). Whether and how VSRs regulate the activity profiles of several miRNAs and thus modulate defense mechanisms during a viral infection have not yet been investigated.

In the study presented, we characterized the interactions of p19 and 2b with the miRNAs 162, 168, and 403 and examined the relevance of these interactions for the activity of these miRNAs. We found the two VSRs showing surprisingly comparable binding profiles with the three miRNAs, despite differently applied RNA-binding modes. However, the affinities of the miRNAs 162, 168, and 403 for the viral proteins vary significantly, and we obtained complementary *in vitro* and, with p19, also *in planta* data demonstrating a correlation between the diverging RNA binding and miRNA-mediated silencing of the *DCL1*, *AGO1*, and *AGO2* mRNAs, which is differently affected by the VSR. Our data propose that diversities in miRNA binding by VSRs, which evolved similarly in different viral contexts, coordinately affect cellular gene expression during the initial stage of an infection. We suggest that this VSR activity promotes infection. On the other hand, we obtained initial hints that plants may counteract this property of VSRs by a modified set of miRNAs.

## RESULTS

### The *Tombusvirus* p19 binds different miRNAs with different affinities.

**FIG 1:**
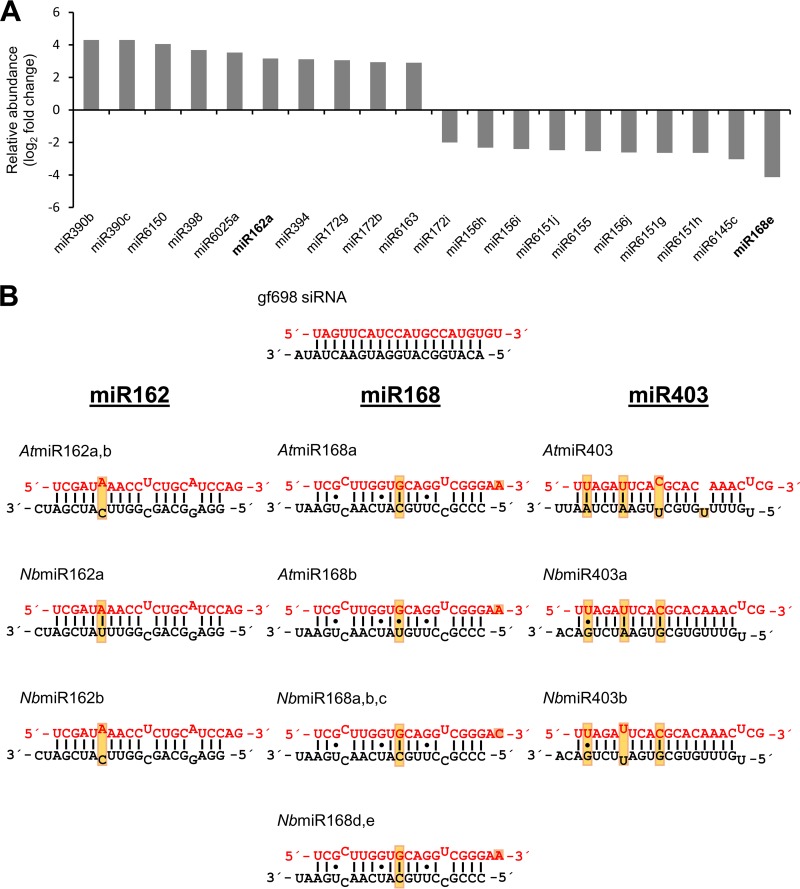
p19 binds miR162 at high preference. (A) Pulldown experiment with CIRV p19. RNA precipitated from BYL by p19-associated beads (see Materials and Methods) was sequenced (full data set recorded as GSE98956), and small RNA sequences were mapped to the annotated Nicotiana tabacum microRNA precursor molecules on http://www.miRBase.org. Shown are 10 miRNAs that were most enriched and 10 miRNAs that were most depleted in the precipitate compared to the input RNAs. The detected miR162 and miR168 isoforms are indicated in bold. All data shown were significant (*P* ≤ 0.05, *n* = 3). (B) Schematic representations of the applied control siRNA gf698 and of the different miR162, miR168, and miR403 isoforms. The miRNAs from *A. thaliana* are annotated in the *A. thaliana* miRBase. The shown miRNAs from *N. benthamiana* derived from a BLAST search of microRNA precursor sequences versus the *N. benthamiana* draft genome at http://www.solgenomics.net (see also Fig. S1). Guide strands are represented in red, and star strands are indicated in black. Dots indicate G⋅U wobbles; mismatched nucleotides are set outward. Positions within an miRNA family with sequence variations are boxed.

As a first approach to characterize the binding behavior of p19 with miRNAs, we performed an RNA pulldown experiment with bead-associated p19 protein of *Carnation Italian ringspot virus* (CIRV) using cytoplasmic extracts of Nicotiana tabacum BY2 cells as the RNA source (see Materials and Methods and below). The precipitated RNAs were subjected to next-generation sequencing (NextGenSeq) of small RNA (sRNA) to identify p19-associated miRNAs. The small RNA sequences were mapped to the annotated Nicotiana tabacum microRNA precursor molecules on http://www.miRBase.org. Interestingly, several miRNAs (10 examples shown in Fig. 1A), among them an isoform of miR162, were found to be enriched in the precipitate compared to input extract. In contrast, some miRNAs (10 examples shown in Fig. 1A), among them an isoform of miR168, were detected at lower abundance within the precipitate. These data suggested that the p19 protein, besides its reported binding properties with siRNAs, has a binding preference for certain miRNAs, one of which is miR162. Other miRNAs, including miR168, were apparently bound at significantly lower efficacy.

10.1128/mBio.00419-18.1 FIG S1  *Nicotiana benthamiana* miRNA precursor 5′-to-3′ sequences and their location in the *N. benthamiana* genome v.0.4.2 at https://solgenomics.net**.** Mature and passenger sequences are highlighted in red (mature) and blue (passenger strands); nucleotides that differ between miRNA isoforms are labeled in green. The search was supported by unpublished sRNA data and facilitated by the fact that miRNA guide strands are highly conserved among dicots (44). Download FIG S1, PDF file, 0.6 MB. Copyright © 2018 Pertermann et al. 2018 Pertermann et al. This content is distributed under the terms of the Creative Commons Attribution 4.0 International license.

To evaluate the p19-miRNA interactions next in a quantitative manner, we performed an electrophoretic mobility shift assay (EMSA [22]) with the purified p19. Besides miR162 and miR168, we decided to test miR403. Like miR162 and miR168, miR403 is supposed to be involved in the antiviral silencing response as well (see the introduction). Considering the intra- and interspecies sequence diversities of these miRNAs, the following RNA isoforms were applied to the assay: from Arabidopsis thaliana (family *Brassicaceae*), miR162, miR168 (two isoforms, a and b), and miR403; and from *Nicotiana benthamiana* (family *Solanaceae*), miR162 (two isoforms, a and b), miR168 (two isoforms, a,b,c and d,e), and miR403 (two isoforms, a and b) (Fig. 1B). Note that the *N. benthamiana* miRNAs and their precursors were newly retrieved from the N. benthamiana genome database (available at https://solgenomics.net/) (see Fig. S1 in the supplemental material). Figure 1B shows that the sequences and duplex structures of the *A. thaliana* miRNAs 162 (*At*miRNAs 162) and *N. benthamiana* miR162b (*Nb*miR162b) are identical and that this is the case with *At*miR168a and *Nb*miRNAs 168d,e. It is apparent that the miR162 and miR168 duplexes are highly conserved between *A. thaliana* and *N. benthamiana* while this is less the case with miR403. Here, identical guide strands are paired with different star strands (Fig. 1B) (44).

**FIG 2:**
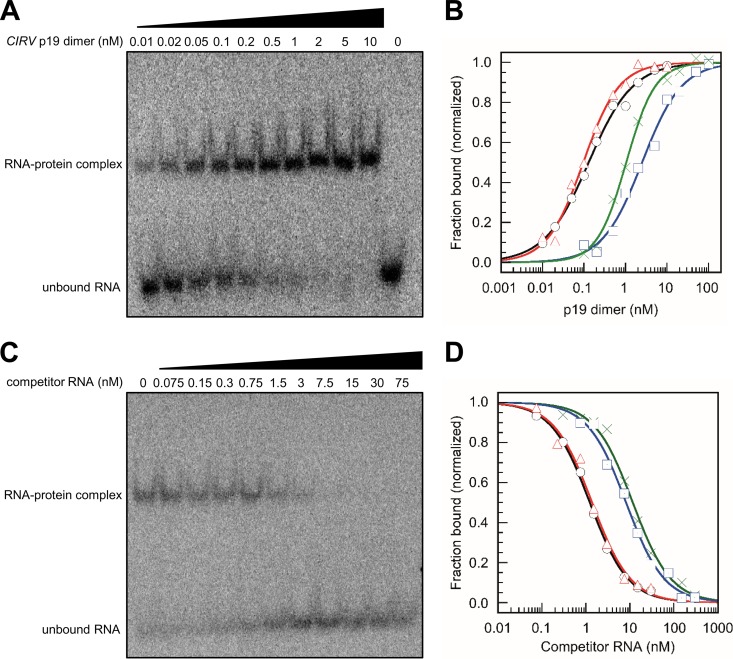
p19 binds different small RNAs at different affinities. (A) Representative gel image of an electrophoretic mobility shift assay (EMSA) showing a direct binding experiment that was performed with 5′-labeled *At*miR162 (≤3 pM) and the indicated amounts of CIRV p19. (B) Representative analyses of binding equilibrium data of CIRV p19 bound to gf698 siRNA (black line with circles), *At*miR162 (red line with triangles), *At*miR403 (green line with crosses), and *At*miR168a (blue line with squares), respectively. Data were fitted to a rectangular hyperbola with cooperativity set variable, equation 1 (see Materials and Methods). Results of the fits are plotted as lines. (C) Representative gel image of an EMSA of a competition assay that was performed with an RNA-protein complex consisting of 5′-labeled gf698 siRNA (≤3 pM) and 0.5 nM CIRV p19 and unlabeled competitor gf698 siRNA at the indicated amounts. (D) Representative analysis of competition data of CIRV p19-siRNA complexes that were outcompeted with the following competitor RNAs: gf698 siRNA (black line with circles), *At*miR162 (red line with triangles), *At*miR403 (green line with crosses), and *At*miR168a (blue line with squares). Data were fitted to equation 3 to calculate *K*_*C*_ values (see Materials and Methods). Results of the fits are plotted as lines.

Figure 2 shows representative gel images (A and C) and binding equilibrium data (B and D), which were obtained in direct binding (A and B) and competition (C and D) experiments with the different miRNAs and an siRNA control, respectively. In the direct binding experiments, p19 protein was titrated against a defined amount of the radiolabeled siRNA and miRNAs, and the dissociation constant *K*_*D*_ was calculated. In the competition experiments, a specific amount of p19 protein complexed with a radiolabeled siRNA was titrated for competition by the unlabeled miRNAs, and the competition constant *K*_*C*_ was calculated (see Materials and Methods; quality of p19 purification is documented in Fig. S2 and S3).

10.1128/mBio.00419-18.2 FIG S2  Purification of CIRV p19. (A) Representative absorption spectrum of purified CIRV p19. (B) Purified CIRV p19 (0.5 µg) separated by SDS-PAGE and stained with Coomassie blue. Download FIG S2, PDF file, 0.3 MB. Copyright © 2018 Pertermann et al. 2018 Pertermann et al. This content is distributed under the terms of the Creative Commons Attribution 4.0 International license.

10.1128/mBio.00419-18.3 FIG S3  Analytical ultracentrifugation of CIRV p19. (A) Equilibrium run at 18,000 rpm, 20°C. (B) Sedimentation velocity at 40,000 rpm, 20°C. (C) Calculation of the sedimentation coefficient. Analysis of data was performed as described in Materials and Methods. The calculated sedimentation coefficient was *s*(app) = 2.74S. From the equilibrium, the corresponding molecular mass was determined to be 46 ± 4 kDa independently of protein concentration (theoretical mass of CIRV p19 monomer, 19.479 kDa). These data confirmed the purified p19 to form a protein dimer (22, 23). Download FIG S3, PDF file, 0.2 MB. Copyright © 2018 Pertermann et al. 2018 Pertermann et al. This content is distributed under the terms of the Creative Commons Attribution 4.0 International license.

The obtained results first confirmed the high binding affinity of p19 to siRNA (Table 1). That is, with the applied siRNA, the measured *K*_*D*_/*K*_*C*_ values were in the range of 0.06 nM, resembling the value of ca. 0.17 nM that was earlier reported with a differently composed siRNA (22). With the tested miRNAs, we observed considerable variations in the binding strength. The measured *K*_*D*_/*K*_*C*_ values of all miR162 versions turned out to be also low and to be in the same range as those with the siRNA (Table 1). In contrast, the *K*_*D*_s of the different miR168 versions were higher by a factor of 40 to 110. With the miR403 versions, we observed species-specific differences in binding. While the *K*_*D*_ was low with *Nb*miR403 (in the range of 0.1 to 0.4 nM), the ratio was ca. 1.4 nM for *At*miR403, i.e., higher by a factor of 4 to 12. Taken together, these data extended the earlier pulldown experiment showing that p19 has a dissimilar binding behavior with different miRNAs: in agreement with earlier observations (35, 42), miR168 shows a weaker binding to p19; in contrast, miR162 and the *Nb*miR403 isoforms display a high, siRNA-like affinity. The *At*miR403, which has a 22-nt star strand (Fig. 1B), was also bound at low affinity.

**TABLE 1:** Binding constants (*K_D_*) determined in direct binding assays of CIRV p19 with different sRNAs and apparent dissociation constants (*K_C_*) determined in competitive binding assays with p19 bound to gf698 siRNA

sRNA	*K*_*D*_ (nM)	Relative *K*_*D*_	*K*_*C*_ (nM)	*K*_rel_ (normalized *K*_*C*_)
gf698 (siRNA)	0.06 ± 0.04	1	0.07 ± 0.03	1
*At*miR162, *Nb*miR162b	0.07 ± 0.03	1.2	0.12 ± 0.05	1.7
*Nb*miR162a	0.14 ± 0.05	1.8	ND^a^	ND
*At*miR168a, *Nb*miR168d,e	2.29 ± 0.78	38.2	1.59 ± 0.35	22.7
*At*miR168b	4.83 ± 1.57	80.5	ND	ND
*Nb*miR168a,b,c	6.57 ± 1.50	109.5	ND	ND
*At*miR403	1.37 ± 0.29	22.8	1.09 ± 0.43	15.6
*Nb*miR403a	0.11 ± 0.02	1.8	ND	ND
*Nb*miR403b	0.37 ± 0.06	6.2	ND	ND

a ND, not determined.

### VSR 2b uses a different RNA-binding mode but shows a comparable miRNA-binding profile to p19.

The observation that the *Tombusvirus* p19 binds regulatory miRNAs involved in antiviral silencing with different affinities raised the question whether this property is also shown by other VSRs. To address this, we purified and tested the full-length VSR 2b of the *Cucumovirus Tomato aspermy virus* (TAV; quality of purification is documented in Fig. S4). As outlined in the introduction, 2b and p19 do not share any primary sequence similarity; however, like p19, 2b was reported to bind siRNAs and miRNAs *in vivo* and *in vitro* (28, 45). Analytic ultracentrifugation measurements showed that the purified TAV 2b is at a monomeric state, independently of its concentration. However, RNA binding causes dimer formation, with one 2b dimer binding two molecules of RNA duplexes (Fig. S5). Accordingly, the RNA-binding mode of TAV 2b clearly differs from that of p19, which exists as a dimer (Fig. S3) and binds one molecule of RNA duplex per dimer (22, 23).

10.1128/mBio.00419-18.4 FIG S4  Purification of TAV 2b. (A) Representative absorption spectrum of purified TAV 2b. (B) Purified TAV 2b gel filtration fractions of ca. 0.1, 0.5, and 0.2 µg of protein were separated by SDS-PAGE and stained with Coomassie blue. The protein bands run at an apparent size of about 12 to 13 kDa; mass spectrometry (matrix-assisted laser desorption ionization–time of flight [MALDI-TOF]) revealed a molecular mass of 11.164 kDa (not shown). (C) Far-UV circular dichroism (CD) spectrum measurements of the same preparation as in panel A indicate a random coil structure and a small helical content (indicated by the arrow) of the RNA-free protein. Download FIG S4, PDF file, 0.2 MB. Copyright © 2018 Pertermann et al. 2018 Pertermann et al. This content is distributed under the terms of the Creative Commons Attribution 4.0 International license.

10.1128/mBio.00419-18.5 FIG S5  Analytical ultracentrifugation of TAV 2b in the absence and the presence of miR162. (A to C) Analyses in the absence of RNA. (D to F) Analyses in the presence of miR162. Isolated protein was used at 2 µM; in complex with miR162, the concentrations were 4 µM TAV 2b and 2 µM miR162. Data monitoring occurred at 230, 260, or 280 nm. (A) Sedimentation equilibrium of TAV 2b (20,000 rpm) yielded an apparent molecular mass of 10.5 kDa. (B) For sedimentation velocity, the sample was measured every 10 min at 40,000 rpm. (C) Data analyses using the program Sedfit resulted in a sedimentation coefficient of *s*(app) = 1.21S. (D) Molecular mass determination of the complex was done at 8,000 rpm and yielded an apparent molecular mass of 51.1 kDa. (E and F) Sedimentation velocity at 40,000 rpm showed an almost homogenous protein/RNA complex with a sedimentation coefficient of *s*(app) = 4.34S (F, black line) together with a small excess of free microRNA [*s*(app) = 2.55S (red line in panel F)]. Download FIG S5, PDF file, 0.3 MB. Copyright © 2018 Pertermann et al. 2018 Pertermann et al. This content is distributed under the terms of the Creative Commons Attribution 4.0 International license.

**FIG 3:**
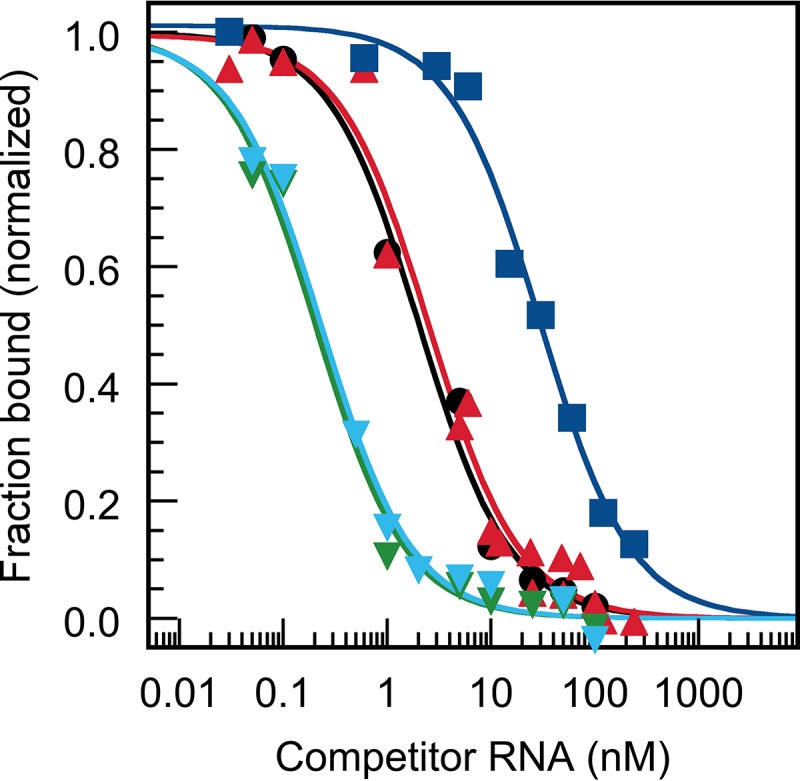
Binding profiles of competition experiments of TAV 2b with miRNAs 162, 168, and 403. Representative analyses of data that were obtained in binding competition experiments (Table 2 and the text present details) with TAV 2b-siRNA complexes. The complexes were outcompeted with unlabeled competitor RNAs (gf698 siRNA [black circles], *At*miR162 [red triangles], *At*miR403 [green inverted triangles], *Nb*miR403b [cyan inverted triangles], and *At*miR168a [blue squares], respectively). The data were fitted to equation 3 (see Materials and Methods).

Purified TAV 2b was tested in EMSA with the miRNAs *At*miR162, *At*miR168a, *At*miR403, and *NbmiR*403a,b, to determine the *K*_*D*_ and *K*_*C*_ values in direct binding and competition assays, respectively. In the direct binding experiment, the TAV 2b was shown to bind all miR162 and all miR403 versions with similar affinities as the control siRNA (*K*_*D*_ values in the range of 0.2 to 0.7 nM) (plots not shown; data are summarized in Table 2). The tested miR168 was observed to associate with detectably lower affinity (*K*_*D*_ of 1.2 nM); however, the discrepancies in the affinities versus the miRNAs 162 and 403 were not as pronounced with 2b as they were in the direct binding experiments with p19 (Table 1). The situation was different in the competition experiments, where the action of the competing RNA duplex takes place on preformed dimeric complexes consisting of 2b and siRNA (Fig. 3). Here, we found that competition was ca. 18 times more effective with miR162 than with miR168; i.e., with miR162, the *K*_*C*_ was determined to be 0.7 nM, while with miR168 it was 12 nM (Table 2). Thus, a direct comparison of the plots of the competition experiments with p19 and 2b (Fig. 2D and 3) revealed that the two unrelated proteins, despite differently applied binding modes, show similar binding performances with the miRNAs 162 (high affinity) and 168 (low affinity). Interestingly, the most effective binding and competition of binding were observed in assays with 2b and the miRNAs 403. In other words, 2b associates with the miRNAs 403 at even higher affinity than the applied siRNA (Table 2). Moreover, in contrast to p19, the 2b protein did not show any species-specific differences in the binding affinities to *At*miR403 and *Nb*miR403 (Table 2; see Discussion).

**TABLE 2:** Binding constants (*K_D_*) determined in direct binding assays of TAV 2b with different sRNAs and apparent dissociation constants (*K_C_*) determined in competitive binding assays with 2b bound to gf698 siRNA

sRNA	*K*_*D*_ (nM)	Relative *K*_*D*_	*K*_*C*_ (nM)	*K*_rel_ (normalized *K*_*C*_)
gf698 (siRNA)	0.68 ± 0.15	1	0.53 ± 0.08	1
*At*miR162, *Nb*miR162b	0.46 ± 0.06	0.7	0.67 ± 0.09	1.3
*At*miR168a, *Nb*miR168d,e	1.24 ± 0.15	1.8	12.1 ± 0.7	22.8
*At*miR403	0.26 ± 0.03	0.4	0.05 ± 0.004	0.09
*Nb*miR403a	0.22 ± 0.03	0.3	0.01 ± 0.006	0.02
*Nb*miR403b	0.26 ± 0.03	0.4	0.02 ± 0.003	0.04

### Sequestration of miRNAs by p19 and 2b controls AGO1/RISC activity.

The fact that viral VSRs bind with different affinities to miRNAs that are involved in the regulation of important antiviral processes raised the question of the biological significance of this finding. To address this, we used first an *in vitro* system based on cytoplasmic extracts of N. tabacum BY2 protoplasts (termed BYL) (46) to prepare AGO proteins enabling the reconstitution of functional RISC. Upon programming with exogenously added siRNAs, RISC specifically cleave target mRNAs (47) or inhibit the replication of viral RNAs (26). *In vitro* reconstitution of RISC is also possible with miRNAs, i.e., miRNA-programmed RISC (miRISC) are active in directing either translation inhibition or cleavage of target mRNAs (47–49).

**FIG 4:**
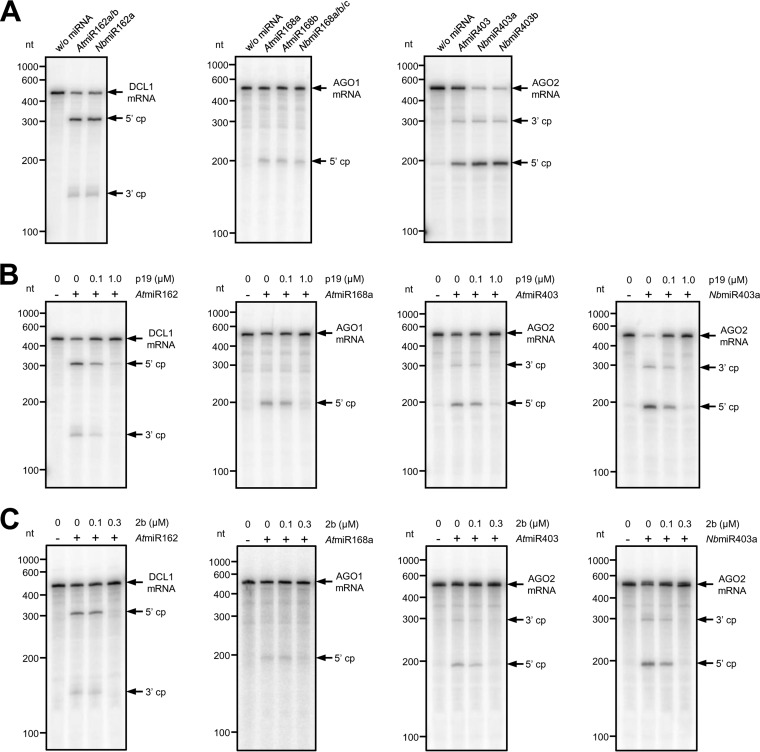
p19 and 2b differently affect microRNA-mediated mRNA cleavage by AGO1/RISC *in vitro*. (A) *NbAGO1* mRNA was translated in BYL in the presence of different, indicated miR162, miR168, or miR403 isoforms to form miRISC. Labeled mRNA fragments containing the respective miRNA target sites were added and analyzed for RISC-mediated cleavage by denaturing PAGE. The target RNA and cleavage products (cp) are indicated by arrows. (B) Experiment analogous to that in panel A with AGO1/miRISC containing *At*miR162, *At*miR168a, *At*miR403, or *Nb*miR403a but performed in the presence of different indicated amounts of p19. (C) Experiment analogous to that in panel B but performed in the presence of different indicated amounts of 2b.

In the first experiment, we tested the miRNAs with their target mRNAs in the BYL. To reconstitute the miRISC in a *Solanaceae* context, we decided to apply AGO1 from *Nicotiana benthamiana* (AGO1-1L [49]). In several plant species, including *N. benthamiana*, AGO1 is well documented to be involved in miRNA-mediated RISC activity (35, 39, 49, 50). Moreover, AGO1 is known to incorporate preferentially the guide strands of small duplex RNAs that initiate with a 5′ uracil (17, 18, 26); in fact, the guide strands of all applied miRNAs initiate with a 5′ U (Fig. 1B). Thus, *AGO1* mRNA was translated in BYL in the presence of the different miR162, miR168, or miR403 versions to form miRISC. Subsequently, radiolabeled target mRNA fragments that contained the respective miRNA target sites were added and the reaction was analyzed for miRNA-directed AGO1-catalyzed cleavage as reported elsewhere (49). As shown in Fig. 4A, specific miRNA-directed slicing was detectable with all tested miRNAs. However, with both applied miR162 isoforms, the efficiencies of the cleavage reactions (i.e., detectable amounts of the 3′ and 5′ cleavage remnants versus residual amount of target input) were evidently higher than with the miR168 isoforms. With the miR403 isoforms, we observed apparent differences. While with both *Nb*miR403 isoforms the cleavage reactions were efficient, this was not the case with the *At*miR403 (Fig. 4A). Hence, we noticed that the slicing activities of the programmed AGO1/RISC varied with the bound type of miRNA.

Next, we repeated the same experiment with *At*miR162, *At*miR168a, *At*miR403, and *Nb*miR403a but supplemented the reaction mixture with two different concentrations of the purified CIRV p19. As shown in Fig. 4B, the slicing reactions of the AGO1/RISC with *At*miR162 and *Nb*miR403 were reduced at a lower concentration (100 nM) but nearly entirely inhibited at a higher concentration (1 µM) of added p19. With *At*miR168 and *At*miR403, solely the high concentration of p19 caused a considerable inhibition of the slicing reaction (Fig. 4B). In similar experiments, we applied the purified TAV 2b. With a lower concentration of the added VSR (100 nM), we observed an evident reduction of the activities of RISC that contained *At*miR403 or *Nb*miR403 as well as a reproducible, slight (ca. 10%) inhibition of RISC that contained miR162. A nearly complete inhibition of the formation of all types of tested miRISC was detectable when 2b was present at a higher concentration (300 nM) (Fig. 4C). Note that the amounts of miRNAs that had to be included in the assay mixture to monitor differential binding of 2b were 10 times smaller than those with p19. We explained this by the different binding modes of p19 and 2b (see above). In sum, these results closely correlated with the earlier measured *K*_*D*_ and *K*_*C*_ values of p19 and 2b to the different miRNAs (Fig. 2 and 3; Tables 1 and 2). They support the conclusion that the two VSRs recruit different miRNAs with different efficacies and, consequently, interfere with the activity of miRNA-loaded RISC in a different manner by sequestering the miRNAs with different efficacies.

### p19 coordinately affects the activities of miR162, miR168, and miR403 in the early phase of *Tombusvirus* infections.

The above results suggested that the differential effect of p19 on miRNA-mediated mRNA silencing is also pertinent during natural infections. We considered the sequestration of miRNAs involved in antiviral immunity by p19 particularly relevant in the early phase of an infection. Thus, after initial rounds of viral translation and replication, the miRNA/vsiRNA ratio should be high in the infected plant tissue and significant amounts of VSR proteins not yet saturated by DCL/RDR-generated vsiRNAs should be present. To investigate this experimentally, we used the well-established Tombusvirus Cymbidium ringspot virus (CymRSV)-*Nicotiana benthamiana in vivo* system and the availability of a replicative, p19-deleted variant of CymRSV, here termed Cym19stop or Δp19 virus (10, 14, 51). Note that the p19 proteins of CymRSV and CIRV are highly homologous. Previous studies with the CymRSV system revealed that at infection times of >4 days postinoculation (dpi), AGO1 expression is induced in the infected plants, which correlates closely with the accumulation of viral RNA (35, 36, 52). Infections with the p19-expressing wild-type (wt) CymRSV were further shown to induce the expression of miR168, which, in turn, inhibits additional accumulation of AGO1 protein at 5 dpi or later (35). Related to this antisilencing effect of p19, CymRSV infections cause a rapid lethal necrosis in infected plants, whereas plants infected with the less invasive Cym19stop show a recovery phenotype (10, 53). Therefore, the following experiments aimed to evaluate the role of p19 on miRNA-mediated gene expression prior to symptom development, i.e., up to the onset of antiviral silencing (induction of AGO1 expression) and p19-mediated anti-antiviral silencing (induction of miR168 expression) at 4 dpi. In pilot experiments, we confirmed the preceding findings showing that during the initial stage of the infection, Cym19stop replicates at a level that is largely comparable to that of the wt CymRSV (21). At 2 dpi, the level of RNA accumulation of the Δp19 virus in inoculated leaves corresponded to ca. 63% of that of CymRSV; at 4 dpi, it was at ca. 58% of the wt level. With both CymRSV and Cym19stop, the relative amounts of viral RNA molecules increased by 5- to 6-fold between 2 and 4 dpi (Fig. S6A). In close accordance with this, the levels of vsiRNAs were determined to be hardly detectable at 2 dpi while at 4 dpi they were significantly elevated (Fig. S6B).

10.1128/mBio.00419-18.6 FIG S6  Relative accumulation of viral gRNA and virus-derived siRNAs in virus-infected *Nicotiana benthamiana* plants. (A) Relative accumulation of CymRSV or Cym19stop genomic RNAs as measured by RT-PCR in inoculated leaves of *N. benthamiana* at 2 dpi (dark gray bars) and 4 dpi (pale gray bars). All viral gRNA levels differ significantly (*P* < 0.05) between CymRSV- and Cym19stop-infected plants at the same day. At 2 dpi, the Cym19stop gRNA levels were 63% of that of CymRSV. At 4 dpi, the Cym19stop gRNA levels were 58% of that of CymRSV. (B) Northern blot analysis of CymRSV siRNA accumulation in inoculated leaves of *Nicotiana benthamiana*-infected plants at 2 and 4 dpi. One mock-inoculated plant and one CymRSV-infected plant at 10 dpi served as controls. Download FIG S6, PDF file, 0.1 MB. Copyright © 2018 Pertermann et al. 2018 Pertermann et al. This content is distributed under the terms of the Creative Commons Attribution 4.0 International license.

As a first approach to test for p19-mediated differences, we infected *N. benthamiana* plants at the six-leaf stage with genomic CymRSV or Cym19stop RNA; extracted the total RNA from the inoculated leaves at 2 and 4 dpi, respectively; and determined the ratios of the star strands and guide strands of miR162, miR168, and miR403 by reverse transcription-quantitative PCR (qRT-PCR). Because p19 is known to bind sRNA duplexes and to stabilize these *in vitro* and *in vivo* (22, 42), the miRNA */g-strand ratios were taken as indicators of the respective p19 binding activity/*in vivo* effect of p19 on the miRNAs. While the */g-strand ratio with a natural miRNA is theoretically 1, we expected this value to be substantially lower in a biological context, i.e., in the presence of functional AGO proteins that incorporate the miRNA’s guide strands and displace the star strands. As summarized in Table 3, this is indeed what we observed at 2 dpi with the */g-strand ratios of miR168, which, in the infections with CymRSV, as well as with the Cym19stop mutant, were measured to be in the range of 0.12. The */g-strand ratios of miR162 and miR403 were also low (0.2) in the Cym19stop-infected plants; however, in the infections with the wt virus (which expresses p19), these ratios were significantly increased by 60% (miR162) and 115% (miR403), respectively (Table 3).

**TABLE 3:** miRNA */g-strand ratios in *N. benthamiana* leaves that were infected with CymRSV or Cym19stop at 2 and 4 dpi

Day and miRNA duplex	CymRSV	Cym19stop	% increase of */g-strand ratio in CymRSV vs Cym19stop infection
2 dpi			
miR162*/miR162^g^	0.32 ± 0.12	0.2 ± 0.1	~60
miR168*/miR168^g^	0.12 ± 0.02	0.12 ± 0.06	None
miR403*/miR403^g^	0.49 ± 0.11	0.23 ± 0.07	~115
4 dpi			
miR162*/miR162^g^	0.37 ± 0.2	0.14 ± 0.1	~165
miR168*/miR168^g^	0.08 ± 0.04	0.09 ± 0.01	None
miR403*/miR403^g^	0.43 ± 0.17	0.18 ± 0.03	~140

As expected, at 4 dpi, the incremental increases in the stabilities of the duplexes of the miRNAs 162 (165%) and miRNAs 403 (140%) were even more evident in the wt virus infection. In contrast, the */g-strand ratio of the miR168 duplex remained unaffected, even at this later time point (Table 3). Note that in congruence with the data of Várallyay et al. (35), the expression of miR168 was found to be at a slightly higher level in the infections with wt CymRSV, while this was not the case with miR162 and miR403 (Fig. S7). In sum, it was obvious that at both time points postinoculation, the presence of p19 in the wt CymRSV infection resulted in increased duplex stabilities of miR162 and miR403, while this was not the case with miR168. Hence, we concluded that in close agreement with the *in vitro* data, also in *N. benthamiana* plants that were infected with CymRSV at an early stage, p19 had an evident binding preference for miR162 and miR403 over miR168. This was most obvious at 2 dpi.

10.1128/mBio.00419-18.7 FIG S7  Relative accumulation of miRNA guide strands in virus-infected *Nicotiana benthamiana* plants. Shown are the relative miRNA levels of miR162, miR168, and miR403 guide strands at 2 dpi (A) and 4 dpi (B). Experiments were done in three repetitions. Note that the levels of miR168 were detectably higher in leaves infected with the p19-expressing virus (CymRSV) than in Cym19stop-infected leaves. This was not the case with miR162 and miR403. Download FIG S7, PDF file, 0.2 MB. Copyright © 2018 Pertermann et al. 2018 Pertermann et al. This content is distributed under the terms of the Creative Commons Attribution 4.0 International license.

**FIG 5:**
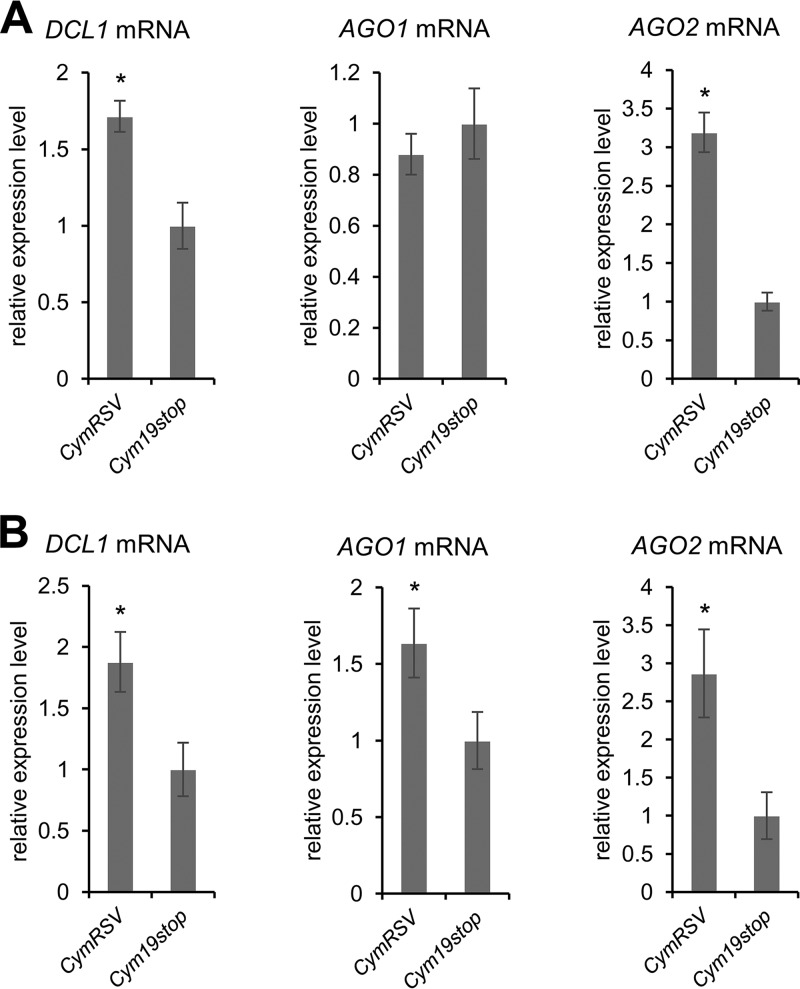
p19 differently alters the mRNA levels of DCL1, AGO1, and AGO2, early in infection. Shown are the measured mRNA levels of *AGO2* mRNA, *DCL1* mRNA, and *AGO1* mRNAs of wt CymRSV- and Cym19stop-infected *N. benthamiana* plants at 2 (A) and 4 (B) dpi, respectively (*n* = 3). The data were statistically analyzed (analysis of variance [ANOVA], F test; *P* > 0.05); significant (*P* ≤ 0.05) differences are indicated by asterisks. Error bars indicate standard deviations.

Using qRT-PCR in total mRNA extractions of infected leaves, we next tested the effects of the CymRSV and Cym19stop infections on the levels of the *DCL1*, *AGO1*, and *AGO2* mRNAs. Thus, at 2 dpi, we observed that *DCL1* and *AGO2* were at significantly higher levels in wt virus- than in Δp19 virus-infected plants. In contrast, the *AGO1* mRNA levels were closely comparable in the two types of infections (Fig. 5A). When we next evaluated the mRNA levels of the analogous experiment at 4 dpi, we observed the same pattern with the *AGO2* and *DCL1* mRNAs. That is, at this later time point of the infection also, the levels of both mRNAs were significantly higher in the wt CymRSV-infected plants (Fig. 5B). However, at 4 dpi, the situation was different with the *AGO1* mRNA, the level of which was also considerably higher in the wt CymRSV-infected plants (Fig. 5B).

**FIG 6:**
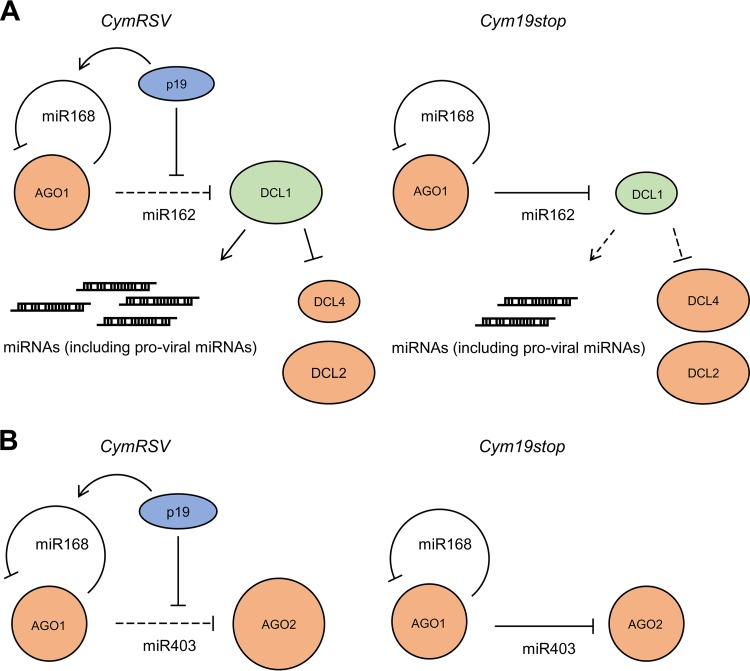
Summarizing model. (A) Effect of p19 on the level of DCL1 early in infection. (Left) Infection with wt CymRSV. By binding of *Nb*miR162 at high affinity, p19 increases the expression of DCL1 and, with this, the expression of miRNAs, some of which (like miR168) may act provirally. High DCL1 levels are suggested to also downregulate DCL4 expression, while the levels of DCL2 remain unaffected (58). miR168-mediated restriction of AGO1 expression (34, 49) is improved by p19 via (i) low association of miR168 (indicated in the work of Várallyay et al. [35] and confirmed here) and (ii) induction of miR168 accumulation (35). (Right) Infection with Cym19stop. In the absence of p19, miR162 downregulates the expression of DCL1, which keeps the miRNA expression levels low and the DCL4 levels high. The miR168-mediated restriction of AGO1 expression is not stimulated. (B) Effect of p19 on the level of AGO2 early in infection. (Left) Infection with wt CymRSV. By binding of *Nb*miR403, p19 increases the expression of AGO2. (Right) Infection with Cym19stop. The AGO2 level remains unaffected (see also Discussion). Antiviral factors are indicated in orange; proviral factors are indicated in green.

These findings suggest that at 2 dpi, with the two viruses replicating at moderate, comparable rates (Fig. S6A), miR162- and miR403-mediated silencing occurred at higher rates in the Cym19stop-infected plants, i.e., in the absence of p19. In contrast, the miR168 activities were the same, irrespective of the presence of the VSR. At 4 dpi, with both viruses replicating at considerably higher levels, the miR162 and miR403 activities were still reduced in the CymRSV-infected plants. However, consistent with the earlier observations (35), *AGO1* mRNA expression was induced at this time point, and this induction was more pronounced in the CymRSV-infected plants correlating with the higher replication rate of the wt virus (Fig. S6A). Taken together, these results suggested that at the early stage of a *Tombusvirus* infection, p19 acts as an efficient caliper of miR162 and miR403 but not of miR168. Accordingly, the formation of functional AGO1/RISC containing miRNA 162 or 403 is effectively inhibited by p19, while this does not occur as efficiently with miR168 (Fig. 6 shows a model; see also Discussion).

## DISCUSSION

miRNAs regulate a wide variety of biological processes, including the measures taken by the plant to counteract and clear pathogen infections (54). The activities of miRNAs are commonly associated with the miRNA’s guide strands that program RISC to silence the expression of target mRNAs by cleavage or translational inhibition (30, 55, 56). As outlined above, virus-encoded VSRs effectively interfere with antiviral RNA silencing. Moreover, studies with several VSRs, including the here-examined *Tombusvirus* p19 and *Cucumovirus* 2b, suggested that an additional feature of these proteins involves the inhibition of miRNA functions (37–41). Thus, VSRs were proposed to interfere with RISC formation by binding to AGO1 (57, 58) or by sequestering miRNAs in a similar way as vsiRNAs (40, 42, 51). Pathogenic effects that were associated with the sequestration of miRNAs by VSRs were most obvious in plants that encode the viral proteins transgenically (42). However, it is apparent that under these conditions, i.e., in the total absence of vsiRNAs, large quantities of ectopically expressed VSR molecules compete for all kinds of small RNA (sRNA) substrates, including also miRNAs. During a natural infection, the situation is more complex. While the initial stage with low viral replication should resemble the transgenic situation by having a high VSR/vsiRNA ratio, this must be reversed at later stages with high viral replication levels and low VSR/vsiRNA and miRNA/vsiRNA ratios. In agreement with this concept, a recent report by Kontra et al. has demonstrated that p19 sequesters plant sRNAs, including miRNAs, effectively in transgenic plants but significantly less effectively in infected plants where systemic symptoms have become apparent (viral infections ongoing for more than 4 days) (50).

This study investigated VSR-miRNA interactions and aimed at understanding how these affect miRNA function in a biological context. Applying the purified p19 and 2b proteins and the miRNAs 162, 168, and 403, we observed that the two VSRs display surprisingly similar miRNA-binding patterns (Tables 1 and 2) despite completely unrelated protein structures and differential RNA-binding modes. While the binding-competent structure of p19 is the dimeric state, our data suggest that in 2b, which, in contrast to earlier studies (28, 59), was applied here as a full-length protein, the RNA-binding capacity evolves from the initial monomer during a dimerization process (see Fig. S5 in the supplemental material; also data not shown). Most importantly, the individual affinities of the miRNAs for the VSRs turned out to be very different. While both proteins associate the miRNAs 162 and *Nb*miR403 in an siRNA-like manner, the *K*_*D*_ values to all miR168 isoforms were significantly higher. Notably, the binding mechanism of the purified 2b to sRNAs was found to be different and the binding affinity was measured to be at a generally higher value as earlier reported (28). We explained this by the here-applied rigorous purification procedure that removes all contaminating nucleic acids (60). With p19, we observed species-specific differences in the sequestration of the *AGO2*-regulating miR403, which could be explained by an increased length of the miRNA’s star strand and other features (discussed below).

We consider the fact that the *Tombusvirus* p19 and the *Cucumovirus* 2b display closely comparable preferences in the binding of miR162, miR403, and miR168 a strong hint for a common biological role of this property, suggesting a parallel functional evolution of the VSRs of the two unrelated viruses. The *in vitro* (Fig. 4) and *in planta* (Table 3; Fig. 5) infection experiments support this notion. Both data sets indicate that the differential binding affinities of the miRNAs for the VSRs that were measured in the biochemical assays indeed affect the three miRNA/mRNA circuits controlling *DCL1*, *AGO1*, and *AGO2* mRNA expression differently. The *in vitro* experiments with the BYL system demonstrated the inhibitory effect of p19 and 2b on miRNA-AGO1/RISC formation most directly. Moreover, these data clearly revealed the close dependence of this effect on the VSR’s miRNA sequestration preferences (Fig. 4B and C). An unrelated but nevertheless interesting finding of these experiments was the observation that in the absence of VSRs, AGO1-catalyzed cleavage of the corresponding mRNA targets was effective with the miRNAs 162 and 403 while this was not the case with the miRNAs 168 (Fig. 4A). We explained this by earlier observations (61), which revealed that besides a 5′-terminal U residue (17, 18) that is present in all miRNAs used in this study (Fig. 1B), the base pairing at the 15th nucleotide is also important for an effective incorporation of miRNAs into AGO1. In contrast to the miRNAs 162 and miRNAs 403, all miRNAs 168 have a mismatch at this position (44). Accordingly, the miRNAs that regulate AGO1 expression also exhibit a lower binding efficacy to the AGO1 protein (61). Our data may be limited by the fact that we tested here exclusively miRNA-AGO1 associations, i.e., the situation may be considerably more complex with other AGOs, which, besides AGO1, were suggested to be involved in miRISC activities as well (17, 18).

The *in vivo* data revealed that miRNA sequestration by VSRs is relevant at the early stage of an infection (Fig. 6), i.e., prior to the emergence of virus-induced symptoms. Hence, we consider the impact of the VSRs on miRNA function to affect the antiviral plant response rather than symptom development. The subsequent sections discuss our findings in the context of the respective regulatory miRNA/mRNA circuits.

### Effective sequestration of miR162; accumulation of DCL1.

Purified p19 and 2b bind all miR162 isoforms with very high affinities, i.e., with *K*_*D*_ values in the picomolar range (Fig. 2 and 3; Tables 1 and 2). The inhibition data of the AGO1/RISC-mediated cleavage of the *DCL1* mRNA that were obtained with the VSRs *in vitro* were in accord with this finding (Fig. 4). Moreover, a considerable stabilization of miR162 duplex and accumulation of *DCL1* mRNA in the *N. benthamiana* plants that were infected with the wt (p19-expressing) CymRSV were detected (Table 3; Fig. 5). This was observed at 2 dpi with a low rate of viral replication, but also at 4 dpi, where viral replication and vsiRNA production were already high (Fig. S6). No data are currently available to suggest that p19 might induce the expression of miR162 (Fig. S7 and data not shown). Hence, we interpret these findings such that at 4 dpi the concentration of p19 molecules that remained unoccupied by vsiRNAs was still sufficient to recruit successfully miR162 and to support *DCL1* mRNA accumulation in the infected plants by inhibiting miRNA/RISC-mediated degradation of the mRNA. Our data are in line with earlier observations by Schott et al. (42), who detected a significant increase in the amounts of DCL1 in p19-transgenic plants, and they also correlate with reports by Qu et al. (7) and Azevedo et al. (58). These colleagues found that in plants infected with *Turnip crinkle virus* (TCV), the level of miR162 was strongly reduced compared to uninfected plants and that this effect depended on the presence of the TCV VSR p38. p38 binds to AGO1, and for that reason the VSR was proposed to reduce the activity of miR162 due to a depletion of active AGO1. The authors further showed that low miR162/high DCL1 levels correlated with lowered expression levels of DCL4 and DCL3, suggesting a close homeostatic DCL interaction network. Thus, our findings are in accord with VSR-mediated upregulation of DCL1 and DCL1-mediated downregulation of the antivirally acting DCL4 and might indeed represent an important measure to counteract antiviral RNA silencing at the early infection stage (model in Fig. 6). Upregulation of *DCL1* mRNA might have another proviral effect considering that DCL1 is the general pri- and pre-miRNA processor (29). This also provides miRNAs that counteract antiviral silencing such as the VSR-induced miR168 (Fig. 6; see also below).

### Noneffective sequestration of miR168; the AGO1 level remains unaffected at the early infection stage.

AGO1 homeostasis is a complex process: in *A. thaliana* and *N. benthamiana*, it was shown to involve a feedback regulatory loop that synchronizes *AGO1* and miR168 expression (34, 49). As already outlined, viral infections generally induce the production of *AGO1* mRNA (35, 39, 57, 62). In turn, the *Tombusvirus* p19, as well as other VSRs like the *Cucumovirus* 2b, specifically induces the accumulation of miR168, counteracting *AGO1* expression (36). In our infection experiments, we indeed detected a slightly induced miR168 production in the wt CymRSV-infected plants, which, however, was not significantly increased from 2 to 4 dpi (Fig. S7). Along these lines, our findings reveal that p19 and 2b have a low affinity for miR168 and that only high concentrations of the VSRs inhibit the incorporation of miR168 into functional AGO1/RISC (Tables 1 and 2; Fig. 4). In fact, *in planta*, we did not detect any impact of p19 on miR168 duplex stability at 2 and 4 dpi and on *AGO1* expression at 2 dpi (Table 3; Fig. 5). However, at the latter time point, the level of wt virus replication apparently exceeded the threshold that leads to a measurable induction of *AGO1* mRNA accumulation. Thus, in close agreement with earlier reports (35, 36, 62), we measured an augmented level of *AGO1* mRNA in the infections with the wt CymRSV at 4 dpi (Fig. 5). In sum, we can state that at the early stage of an infection (2 dpi) and in contrast to the situation with *DCL1* and *AGO2* (see below), the mRNA level of the main microRNA effector, AGO1, is apparently not affected by the presence of p19. This was experimentally further confirmed when we compared the AGO1 protein levels in the leaf material of the two types of infected plants. The amounts of AGO1 were essentially in the same range in the wt and Δp19 virus-infected plants as revealed by Western blotting experiments (Fig. S8).

10.1128/mBio.00419-18.8 FIG S8  Western blot analysis of total protein extracts for AGO1 accumulation. Cym19stop*-* and CymRSV-inoculated leaves of *Nicotiana benthamiana* were homogenized at 2 and 4 dpi and used for protein extraction. *Nb*AGO1 protein was detected using a rabbit antibody raised against two N-terminal peptide sequences of the protein. Actin was detected as a loading control. Note that the *Nb*AGO1 levels do not differ between CymRSV- and Cym19stop-infected plants. Download FIG S8, PDF file, 0.1 MB. Copyright © 2018 Pertermann et al. 2018 Pertermann et al. This content is distributed under the terms of the Creative Commons Attribution 4.0 International license.

These findings were also important for another reason, as they excluded the possibility that the significantly higher concentrations of the *DCL1* and *AGO2* mRNAs that were measured in the CymRSV-infected plants were caused by differential recruitment of the miRNAs 162 and 403 by different quantities of AGO1/RISC. Rather, these data supported the conclusion that the increased mRNA levels were indeed caused by the high-affinity sequestration of the miRNAs 162 and 403 by p19.

### Evolution of miR403; an antiviral mechanism?

While miRNAs 403 are absent in monocots, in several dicots, including *A. thaliana* and *N. benthamiana*, they were indicated to target and regulate the expression of *AGO2*, as well as of the closely *AGO2*-related *AGO3* (44). Sequence data suggest a very recent evolution of miRNA 403 (44); in fact, the miRNAs 403 of *A. thaliana* and *N. benthamiana* show apparent differences (Fig. 1B). The *At*miR403 displays an extended length of the star strand, and in consequence, one nucleotide forms a bulge, which may introduce a particular bend in the RNA duplex. The *Nb*miRNAs 403 generally show a high proportion of perfectly base-paired regions, and *Nb*miR403 isoform b contains one mismatch (Fig. 1B). While these differences may explain the significantly lower affinity of *At*miR403 (and the slightly reduced affinity of *Nb*miR403b) for p19, the 2b VSR does not show such discrimination. Accordingly, p19 binding of the *Nb*miR403 molecules and 2b binding of all miRNAs 403 occur at affinities that are in the same range as or even exceed those of siRNAs (Tables 1 and 2; Fig. 2 and 3). Expression of the VSRs thus should prohibit very effectively the incorporation of these miRNAs into AGO1/RISC and, in consequence, should promote *AGO2* mRNA expression (Fig. 6). In fact, this is what we observed in the *in vitro* slicing experiments with the purified CIRV p19 and TAV 2b (Fig. 4) and in the *in vivo* infection experiments with the p19-expressing CymRSV wt virus (Fig. 5). With regard to the *in vivo* data, it is important to note that *Tombusvirus*-infected *N. benthamiana* plants were recently reported to show an increased accumulation of AGO2 at late infection stages (≥6 dpi), irrespective of whether the viruses expressed p19 or not (52). While this suggests a general viral infection- or replication-associated induction of AGO2, similar to that of AGO1, our data revealed significant differences in the *AGO2* mRNA levels at the early infection stage of 2 dpi where the replication levels of CymRSV and Cym19stop are closely comparable (Fig. 5 and S6). We considered these data a strong hint for a p19-mediated hijacking of the *Nb*miRNAs 403.

The biological relevance of these observations is not as obvious as with the high-affinity sequestration of miRNA 162 by p19 and 2b discussed above. Recent evidence clearly indicates that AGO2 acts antivirally, exerting slicing as well as translational repression activities. With AGO1 regulating *AGO2* expression via miR403, AGO2 is accordingly assumed as a “second layer” of virus-restricting RNA silencing, which functions either independently or in a nonredundant manner together with AGO1 and/or AGO5 (26, 63–65). Along this line, our data may reveal an interesting relationship between binding affinities of the miRNAs 403 to the viral VSRs and plant susceptibility to an infection. As explained above, *A. thaliana*, which is not permissive for CymRSV and other tombusviruses, expresses an miR403 that has a considerably lower affinity for p19 than the miRNAs 403 of *N. benthamiana*, which is permissive (Table 1). *Cucumber mosaic virus* (CMV), a close relative of TAV, infects *A. thaliana* as well as *N. benthamiana* (57), and as explained above, the 2b VSR binds all types of miR403 with high affinity (Table 2). Thus, in view of the suspected recent development of miR403 (44) and its variability, high-affinity binding of miR403 may be considered a plant species-specific counter-counterdefense mechanism. Accordingly, a highly interesting topic for future studies involves correlating the susceptibility of plants to infection and the evolution of the properties of miRNAs 403 to act as binding partners of viral VSRs.

In summary, our study supports the view that evolution of miRNAs and their targets is a crucial constituent of plant evolution (66). Thus, adaptation to changing environmental conditions such as the exposure to new or modified types of viruses may occur by the selection of plant species expressing a modified arsenal of miRNAs.

## MATERIALS AND METHODS

### Plasmid constructs.

For protein expression, the CIRV p19 open reading frame (ORF) was cloned via PCR into the pGEX-6P-1 vector (GE Healthcare, United Kingdom) (see Table S1 in the supplemental material for PCR primers). To generate the CIRV p19 alanine variants, site-directed mutagenesis was performed by PCR. The full-length ORF of 2b of TAV (NCBI sequence identifier [ID] AJ320274.1) was received after synthesis in a pEX-A2 backbone (Eurofins, Germany). For protein synthesis, the TAV 2b ORF was cloned via PCR into the pETSUMO vector (Life Technologies, CA). Successful cloning was verified via sequencing (Seqlab, Germany).

10.1128/mBio.00419-18.9 TABLE S1  DNA oligonucleotides used in this study. Download TABLE S1, PDF file, 0.2 MB. Copyright © 2018 Pertermann et al. 2018 Pertermann et al. This content is distributed under the terms of the Creative Commons Attribution 4.0 International license.

### Protein expression and purification.

Glutathione *S*-transferase (GST)–p19 fusion protein was expressed in Escherichia coli BL21(DE3) RIPL cells after induction with 1 mM isopropyl-1-thio-β-d-galactopyranoside (IPTG; Roth, Germany). For cell lysis, bacterial pellets were resuspended in lysis buffer 1 (50 mM Tris-Cl, pH 7.5, 100 mM NaCl, 1 mM dithiothreitol [DTT], 100,000 U/ml lysozyme; Sigma-Aldrich), incubated on ice, and lysed with a French press. For purification of the fusion protein, the soluble fraction was loaded onto a GSTrap column (5 ml; GE Healthcare). After washing with phosphate-buffered saline, the column was equilibrated with cleavage buffer (50 mM Tris-Cl, pH 7.5, 100 mM NaCl, 1 mM DTT), and cleavage was performed with 100 U of PreScission protease (GE Healthcare) at 4°C overnight. Following elution with cleavage buffer, the p19 was additionally purified via a Resource Q anion exchange column (GE Healthcare), concentrated to ~40 µM, transferred to storage buffer (10 mM Tris-HCl, pH 7.5, 150 mM NaCl, 1 mM EDTA, 1 mM DTT, 50% [vol/vol] glycerol) using ultrafiltration, and stored at −20°C. His-SUMO-2b fusion protein was expressed in E. coli BL21(DE3) RIPL cells that were grown at 37°C and induced by 0.4 mM IPTG. After induction, the cells were grown at 25°C overnight. For lysis, the cells were resuspended in lysis buffer 2 (50 mM potassium phosphate, pH 7.6, 500 mM NaCl, 1 mM DTT containing 1 mM phenylmethylsulfonyl fluoride [PMSF], and protease inhibitor; Sigma-Aldrich), pretreated at 4°C with lysozyme (1.5 mg/g cells; Sigma-Aldrich), and lysed with a French press. The lysate was centrifuged at 40,000 rpm and 10°C, and the soluble fraction containing the TAV 2b fusion protein was directly loaded onto a HisTrap FF column (5 ml; GE Healthcare). After washing with buffer 2, the protein was eluted with buffer 3 (50 mM sodium phosphate, pH 7.6, 500 mM NaCl, 500 mM imidazole, 1 mM DTT) and dialyzed against buffer 4 (10 mM Tris-Cl, pH 7.6, 25 mM NaCl, 1 mM DTT). After filtration (0.22 µm), the solution containing the fusion protein was applied to an anion exchange chromatography column (Hi Prep Q HP 16/10; GE Healthcare). Elution of the target protein was performed using a linear gradient of buffer 5 (10 mM Tris-Cl, pH 7.6, 250 mM NaCl, 1 mM DTT). The His-SUMO tag was then removed by SUMO protease cleavage. Afterward, the protein was directly applied to a heparin affinity chromatography column (2 × 5 ml HiTrap Heparin HP; GE Healthcare), washed with buffer 6 [50 mM Tris-Cl, pH 7.6, 100 mM NaCl, 1 mM tris(2-carboxyethyl)phosphine (TCEP)], and eluted with a linear gradient of buffer 7 (50 mM Tris-Cl, pH 7.6, 1 M NaCl, 1 mM TCEP). Fractions containing the target protein were collected and concentrated using Vivaspin 20 (Sartorius) with a molecular mass cutoff of 5,000 Da. The concentrated protein solution was supplied to a final size exclusion chromatography plate on a G25 column for removal of impurities and rebuffering with buffer 6. The final concentration was determined as 2.67 mg/ml. Samples were quickly frozen and stored at −80°C.

### Analytic ultracentrifugation.

Sedimentation velocity and equilibrium measurements were performed at 20°C at 128,794 × *g* (40,000 rpm) and 5,152 × *g* (8,000 rpm), respectively, using an XL-A analytical ultracentrifuge (Beckman Instruments, CA), double-sector cells, and an An50Ti rotor. Analyses were conducted at protein concentrations of 1.2 to 13 µM (CIRV p19) or 1 to 23.8 µM (TAV 2b) in buffer D. The data obtained were analyzed using Sedﬁt.

### *In vitro* transcription.

*NbAGO1-1L* mRNA and CIRV *p19* mRNA were synthesized in the presence of monomethylated cap analogue m^7^GP_3_G (Jena Biosciences, Germany) from SwaI-linearized plasmid constructs described by Gursinsky et al. (49) using SP6 RNA polymerase (Thermo). Transcripts encoding the firefly luciferase mRNA were generated by SP6 RNA polymerase from the XhoI-linearized plasmid pSP-luc(+) (Promega). Transcription reactions and subsequent treatment of the transcripts were performed by using standard procedures. To generate target RNAs for miRNAs 162, 168, and 403, cDNA fragments of the *A. thaliana DCL1* ORF, the *N. benthamiana AGO1-1L* ORF, and the *N. benthamiana AGO2* 3′ untranslated region (UTR) containing the respective miRNA target site were amplified by PCR with the T7 promoter sequence included in the forward primer. Transcription of the target RNAs was performed from the PCR products by T7 RNA polymerase in the presence of 0.5 µCi/µl [α-^32^P]CTP (3,000 Ci/mmol) under standard conditions.

### Analysis of p19-bound miRNAs.

The miRNA pulldown was performed with the microRNA detection kit of New England BioLabs (NEB) using 20 µg of total RNA from BY2 lysate (BYL; see below) and 7 U of CIRV p19 (New England BioLabs, MA) under the conditions recommended by the manufacturer. The precipitate was digested with 20 µg of proteinase K (Thermo) at 0.5% (mass/vol) of sodium dodecyl sulfate (SDS), and the RNAs were obtained by phenol-chloroform extraction and ethanol precipitation. NextGenSeq was performed with an Illumina Highscan at the Core Unit DNA Technologies of the University of Leipzig, Germany.

### Bioinformatic analysis of NextGenSeq data.

Input and precipitation samples were taken from three individual lots of cells. Raw reads were adapter clipped using cutadapt (v1.12) and quality filtered (-q 20) and trimmed (-l 15) by Sickle. Reads were mapped onto a set of known rRNA genes using Bowtie (v1.1.2) (-n 3). Unmapped/rRNA free reads were mapped onto a combination of the miRNA hairpins from the miRBase (v21) (http://www.miRBase.org/) and snoRNAs from the Plant snoRNA Database (http://bioinf.scri.sari.ac.uk/cgi-bin/plant_snorna/home) using Bowtie. Mapped reads were counted using a customized R script. The differential expression analysis was performed with Bioconductor package edgeR (v3.12.1). miRNAs were declared differentially expressed if the false discovery rate (FDR) was below 0.05. The coordinates of the *N. tabacum* mature miRNAs and their hairpins were extracted from the miRBase data file (ftp://miRBase.org/pub/mirbase/21/miRNA.dat.gz). Error bars were calculated by the equation

$$\sqrt{{\text{standard error}}^{}\;{\text{(p}19}^{-}\;{\text{V}}^{\text{+}}{\text{)}}^{\text{2}}{\text{ + standard error }}^{}{\text{(p}19}^{+}\;{\text{V}}^{\text{+}}{\text{)}}^{\text{2}}}$$

### Origin, generation, and modification of examined sRNAs.

The gf698 siRNA used as a control was earlier described by Iki et al. (47). It targets the mRNA of green fluorescent protein (GFP). The sequences of the applied miRNAs from Arabidopsis thaliana were derived from the miRBase database (http://www.miRBase.org). For the identification of the miRNAs 162, 168, and 403 from *Nicotiana benthamiana*, precursor sequences of the Nicotiana tabacum miR162 and miR168 and of the Solanum lycopersicum miR403 were blasted against the *N. benthamiana* draft genome sequences at http://www.solgenomics.net. The *N. benthamiana* miRNAs (Fig. 1B) were deduced from the identified miRNA precursor sequences (Fig. S1); their fold was predicted by Mfold. RNA oligonucleotides (Table S2) to generate the here-examined sRNAs were purchased from Biomers (Germany). Since phosphorylation was earlier shown to increase the binding affinity of sRNAs by CIRV p19 (22), all oligonucleotides were 5′ phosphorylated using T4 polynucleotide kinase (Thermo) and standard procedures. For radiolabeling, 25 µCi of [γ-^32^P]ATP (3,000 Ci/mmol) was added to the phosphorylation reaction mixture. For sRNA annealing, the phosphorylation reactions were stopped by the addition of EDTA and reactions of two complementary oligonucleotides were combined. After heating at 94°C for 3 min, the mixture was cooled at a rate of 3°C/3 min to 25°C. Hybridized RNA duplexes then were purified with Illustra microSpin G-25 columns (GE Healthcare) as suggested by the manufacturer.

10.1128/mBio.00419-18.10 TABLE S2  RNA oligonucleotides used in this study. Download TABLE S2, PDF file, 0.1 MB. Copyright © 2018 Pertermann et al. 2018 Pertermann et al. This content is distributed under the terms of the Creative Commons Attribution 4.0 International license.

### Determination of binding affinities.

For direct measurement of the binding affinities, radiolabeled siRNA (≤3 pM for CIRV p19; ≤30 pM for TAV 2b) was incubated with different concentrations of the purified protein in binding buffer (20 mM Tris-Cl, pH 7.5, 100 mM NaCl, 1 mM EDTA, 1 mM DTT, 0.02% Tween 20) at 24°C for 1 to 2 h. For competition experiments, the unlabeled but phosphorylated miRNAs were titrated against radiolabeled gf698 siRNA (≤3 pM for CIRV p19; ≤30 pM for TAV 2b) that had been bound to a fixed concentration of protein (0.5 nM CIRV p19 dimer or 1.9 nM TAV 2b monomer) in binding buffer overnight. The samples were mixed at 0.25% (vol/vol) with loading dye (50 mM Tris-HCl, pH 7.5, 10 mM EDTA, 0.002% bromophenol blue, 0.002% xylene cyanol, 50% [vol/vol] glycerol) and analyzed by PAGE on a native 6% Tris-borate-EDTA (TBE) gel. The protein-bound and free RNAs were detected by phosphorimaging (Storm 860; Molecular Dynamics) and quantified by ImageQuant software (GE Healthcare). All measurements were done minimally in triplicate.

Since CIRV p19 always forms a dimer (Fig. S3), the fractions of p19-bound radiolabeled gf698 were plotted versus the free protein dimer concentrations and fitted to a rectangular hyperbola for binding constant determination in direct binding reactions.

(1) $$S_{n}=\frac{D_{t}^{n}}{K_{D}+D_{t}^{n}}$$

*S*_*n*_ is the bound sRNA fraction, *D*_*t*_ is the total protein dimer concentration, *K*_*D*_ is the binding constant, and *n* is the cooperativity.

In contrast to earlier reports (28, 59) in which experiments were performed with a truncated version of TAV 2b, the full-length TAV 2b was shown here to be present as a monomer in the absence of RNA, which forms dimers while binding to small RNAs at a ratio of one sRNA duplex per monomer (Fig. S5). To consider this, the fractions of bound radiolabeled gf698 were plotted versus free protein dimer concentrations and fitted to an adapted rectangular hyperbola for binding constant determination in direct binding assays:

(2) $$S_{n}=\frac{4\cdot D_{t}+K_{D}-\sqrt{K_{D}{}^{2}+8\cdot D_{t}\cdot K_{D}}}{4\cdot D_{t}}$$

For competition reactions, the fractions of bound radiolabeled gf698 were plotted versus free competitor RNA concentrations and fitted to the following equation:

(3) $$S_{n}=\frac{1}{2\cdot R_{t}}\cdot \left[K_{D}+\frac{K_{D}}{K_{C}}\cdot C_{t}+P_{t}+R_{t}-\sqrt{{\left(K_{D}+\frac{K_{D}}{K_{C}}\cdot C_{t}+P_{t}+R_{t}\right)}^{2}-4\cdot R_{t}\cdot P_{t}}\right]$$

*R*_*t*_ is the total concentration of radiolabeled gf698, *C*_*t*_ is the total competitor RNA concentration, *P*_*t*_ is the total protein concentration (for CIRV p19, dimer concentration; for TAV 2b, monomer concentration), and *K_C_* is the apparent dissociation constant of the competitor RNA.

### Cell culture and preparation of BYL.

Nicotiana tabacum BY2 cells were cultured at 23°C in Murashige-Skoog liquid medium (Duchefa, Netherlands). Evacuolated BY2 protoplasts were obtained by Percoll gradient centrifugation, and cytoplasmic extract (BYL) was prepared as described earlier (46, 67).

### *In vitro* slicer assay.

To generate miRNA-programmed AGO1/RISC *in vitro*, *NbAGO1-1L* mRNA was *in vitro* translated in 50% (vol/vol) BYL under the previously described conditions (26, 67). Briefly, 0.5 pmol *AGO1* mRNA was translated in a 20-µl reaction mixture in the presence of 100 nM miRNA duplex. For slicer assays in the presence of VSR, 5′-phosphorylated miRNA duplexes (100 nM in the case of the p19 experiments and 10 nM in the case of the 2b experiments) were used and the indicated amounts of purified VSR were applied to the reaction mixture at the same time point as the respective miRNA and the *AGO1* mRNA. After 2.5 h at 25°C, 2 µg of firefly luciferase (competitor) mRNA and the ^32^P-labeled target RNA (50 fmol) were added, and the cleavage reaction was performed for 15 min. Total RNA was isolated from the reaction mixture by treatment with 20 µg proteinase K in the presence of 0.5% SDS for 30 min at 37°C, followed by chloroform extraction and ethanol precipitation. ^32^P-labeled products were separated on 5% TBE polyacrylamide gels containing 8 M urea and visualized by phosphorimaging (Storm 860; Molecular Dynamics). All measurements were performed minimally in triplicate.

### Plant infection experiments, RNA extraction, and qRT-PCR of miRNAs and mRNAs.

The quantification of miRNA expression by qRT-PCR was carried out according to the protocol of Pantaleo et al. (68) with some modifications. One microgram of total RNA was polyadenylated using the poly(A) tailing kit (Life Technologies) according to the manufacturer’s instructions. The RNA was precipitated by ethanol and reverse transcribed with Moloney murine leukemia virus (M-MLV) reverse transcriptase (Life Technologies) and 0.5 µg poly(T) adapter (Table S1). For the amplification of miRNAs, a universal 3′-adapter reverse primer set was used (Table S1), and the forward primer was designed based on the specific miRNA sequence. The relative expression was calculated based on the comparative threshold cycle (*C*_*T*_) (2^−ΔΔ*CT*^) method as described previously (69). The PCR mix (10 µl) contained 5 µl PowerSYBR Green master mix (Applied Biosystems, Life Technologies), 0.25 µM (each) primer, and 1 µl of cDNA diluted 1:100. Cycling conditions for all primer pairs consisted of initial denaturation at 95°C for 10 min, followed by 45 cycles at 95°C for 15 s and 60°C for 1 min. 5.8S rRNA and U6 were used as housekeeping genes.

For the quantification of expression levels of target genes, primers for qRT-PCR (Table S1) were designed using the software Primer Express 3.0 (Life Technologies) in correspondence to predicted cleavage sites. First-strand cDNA synthesis was performed using 2 µg of total RNA treated with DNase and the high-capacity cDNA reverse transcription kit (Life Technologies). The relative expression was calculated based on the comparative *C*_*T*_ (2^−ΔΔ*CT*^) method. The PCR mix (10 µl) contained 5 µl of PowerSYBR Green master mix (Life Technologies), 0.25 µM (each) primer, and 1 µl of cDNA diluted 1:10. Cycling conditions for all primer pairs consisted of initial denaturation at 95°C for 10 min, followed by 40 cycles at 95°C for 15 s and 60°C for 1 min. PCR was performed in triplicate, and specific annealing of the primers was controlled on dissociation kinetics performed at the end of each PCR run. As an endogenous housekeeping gene, cyclophilin (CPH) was used as reported by Havelda et al. (62). Relative quantification of CymRSV genomes was carried out using primers designed on viral RNA-dependent RNA polymerases to exclude the amplification of subgenomic mRNAs (Table S1) and following the same qRT-PCR parameters reported above for the target genes. Data were expressed as the mean and standard error calculated for three biological replicates, and the results were statistically analyzed using Student’s *t* test.
