# Supplementary material for: A Viral Suppressor Modulates the Plant Immune Response Early in Infection by Regulating MicroRNA Activity
Source: mBio. 2018 Apr 24;9(2):e00419-18. doi: 10.1128/mBio.00419-18 (PMC5915741; doi:10.1128/mBio.00419-18)
Supplement: FIG S1 [file mbo002183848sf1.pdf]

Fig. S1

>nbe-miR403a precursor
AAAGAGTCATATTTCACGTTTGTGCGTGAATCTGACAACCCTTTTTATCATTTTTATCTCATGGGTTGTGTTTGTTAGATTCACGCACAAACTCG
CAATATGTCTTTTCT
Location: >Niben101Scf00793Ctg034:3501-4700

>nbe-miR403b precursor
TCTGAAAGAGGCATATTTCACGTTTGTGCGTGATTCTGACAACCATTTTTCATCATTAAATTTATTTTTATCTCATTGGTTGTGTTTGTTAGATTC
ACGCACAAACTCGTAATATGACTTTTCCTCATCT
Location: >Niben101Scf06412Ctg035:201-1400

>nbe-MIR162a precursor
TGGTTGGTTAAGAACACTGGAGGCAGCGGTTTATCGATCTGTTCCCTGAAAAGCGATAAACAAAATATAGCAACAGGAATCGGTCGATAAACC
TCTGCATCCAGCGGTTAACCCCCTTCT
Location: > Niben101Scf11303Ctg014:3301-4500

>nbe-MIR162b precursor
TTCTTTTGGTTGGTTAAAAACACTGGAGGCAGCGGTTCATCGATCTGTTCCCTGAAAGGCGATAACAAAAATATAGCAACAGGAATCGGTCGA
TAAACCTCTGCATCCAGCGCTTAACCCCCTTCTCTCT
Location: >Niben101Scf01764Ctg011:2801-4000 (-1)

>nbe-MIR168a precursor
TGTCTCTAATTCGCTTGGTGCAGGTCGGGACTAATTCGCCGGCGACGGCGGCAATCACGACGACGGTGATTGTTATTTAATGGAGTTTAGAC
GTACGAAGTTATCAACATTTTTTTGTTTTGCAGCGAAATTTGTCCCGCCTTGCATCAACTGAATTGGAGACTGC
Location: >Niben101Scf10054Ctg018:1201-2400

>nbe-MIR168b precursor
CTCTAATTCGCTTGGTGCAGGTCGGGACTAATTCGCCGGCGGCGGCAATCACGACGGCGGTGATTGTTATTAAATGGAGTTAAGATGTACGA
AGTTATCAACTTTTTTTGTTTTGCGGCGAAATTTGTCCCGCCTTGCATCAACTGAATTAGAGACTGC
Location: > Niben101Scf10307Ctg006:2101-3300

>nbe-MIR168c-1 precursor
GTCTCTCATTCGCTTGGTGCAGGTCGGGACCTGCTTTGTCGGCGACAATGACGTCAGCTGACGGTGACGGTGGCATATCGATAGATATACAT
GTTTATGACGAAGTTTGGGTCCCGCCTTGCATCAACTGAATCGGAGACTGCGGCGAAT
Location: >Niben101Scf04636Ctg025:701-1900

>nbe-MIR168c-2 precursor
GGTCTCTCATTCGCTTGGTGCAGGTCGGGACCTGTCTCGCCGGCGACAATGACGTCAGCTGACGGTTACGGTGCCGTATCGATAGATATACA
TGTTGTTTATGACGAAGTTTGGGTCCCGCCTTGCATCAACTGAATCGGAGACTGCGGT
Location: >Niben101Scf07765Ctg021:44501-45800 (-1)

>nbe-MIR168d precursor
GGTCTCTAATTCGCTTGGTGCAGGTCGGGAACTGACTCGCCGGTGACGCTGTCCGCGCCGGAATCGGCGCCCTTAGTTTCATTGATATTTTG
TTTACATGTACCGATGTTTAGAGAGCTCTCTTTTGGACTTATTTGTTAGTAATTTTTGTTGTTGAAAATAACTTGATATATGTATAGCTAAGTAGA
GAGTGTTTACCCACGGTTAGTTAAGTAATAGATACTATGTAGAGAGTGTTTACCACGGTTGGTTAGGTAATGGATATGTAGAGAGTTCATGTTT
TTAATAGATTAGTTATGAATGAAAGTGCGCCTCGTAAATGGCTAAGTTTGTTCCCGCCTTGCATCAACTGAATTGGTGACCGC
Location: >Niben101Scf08513Ctg014:21401-22800 (-1)

>nbe-MIR168e precursor
CTCTAACTCGCTTGGTGCAGGTCGGGAACTGACTCGCCGGTGACGCTGTCCGCGCCGGAATCGGCGCCCTTAGTCTCAATCATTTTTTGTTT
ATATGCATCGATATTCAGAGAGCTCTCATTAGGACTTATTTGTTAGTAATTTTTTGTTGTTGAAATTAACTTGATATATGTATAGATAAGTAGAGA
GTGTTTACCCCCGGTTGGTTAAGTAATAGATATGTAGAGACTGTTTGCCCACGGTTGGTTCCACGGTTGGTTAAGTAATGGATATGTAGAGAG
TTCAATTTTTTTTAATAGATTAGTTATGAATGAAATTGCGCCTCGTCAATGGCTAAGTTTGTTCCCGCCTTGCATCAACTGAATCGGTGACCGC
Location: >Niben101Scf07340Ctg008:19901-21400

Fig. S1. *Nicotiana benthamiana* miRNA precursor 5'-to-3' sequences; their location in the *Nb* genome v.0.4.2 at https://solgenomics.net/. Mature and passenger sequences are highlighted in red (mature) and in blue (passenger strands); nucleotides that differ between miRNA isoforms are labeled in green. The search was supported by unpublished sRNA data and facilitated by the fact that miRNA guide strands are highly conserved among dicots (44).
