# Supplementary material for: A Viral Suppressor Modulates the Plant Immune Response Early in Infection by Regulating MicroRNA Activity
Source: mBio. 2018 Apr 24;9(2):e00419-18. doi: 10.1128/mBio.00419-18 (PMC5915741; doi:10.1128/mBio.00419-18)
Supplement: FIG S2 [file mbo002183848sf2.pdf]

Fig. S2

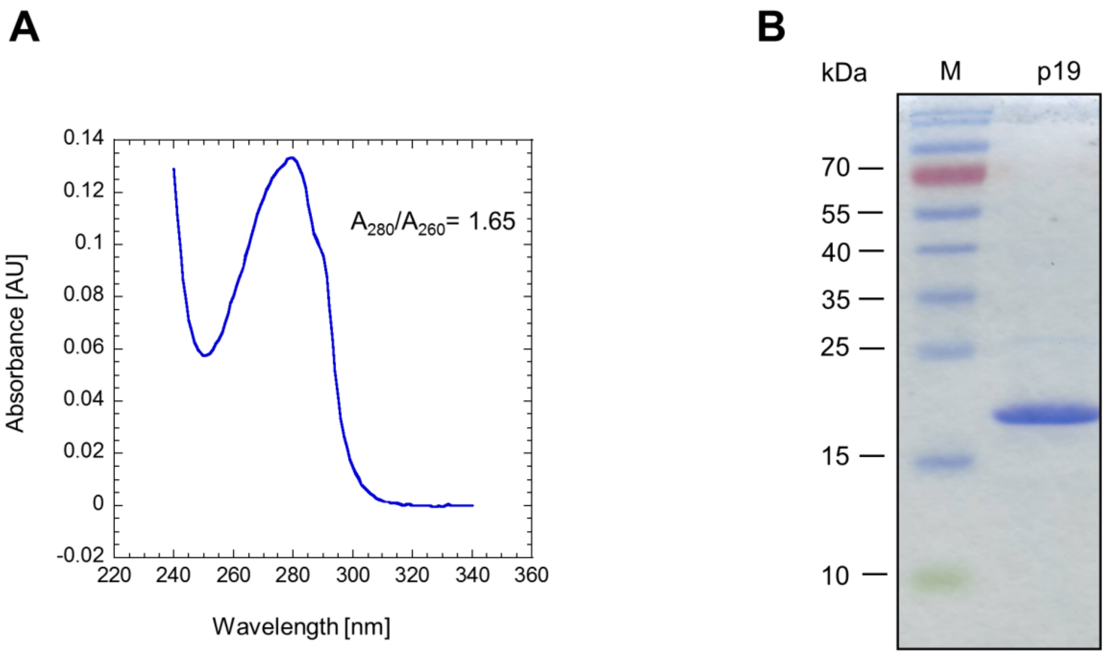

Fig. S2. Purification of *CIRV* p19. (A) Representative absorption spectrum of purified *CIRV* p19. (B) Purified *CIRV* p19 (0.5 µg) separated on SDS PAGE and stained with coomassie blue.
