# Supplementary material for: A Viral Suppressor Modulates the Plant Immune Response Early in Infection by Regulating MicroRNA Activity
Source: mBio. 2018 Apr 24;9(2):e00419-18. doi: 10.1128/mBio.00419-18 (PMC5915741; doi:10.1128/mBio.00419-18)
Supplement: FIG S3 [file mbo002183848sf3.pdf]

Fig. S3

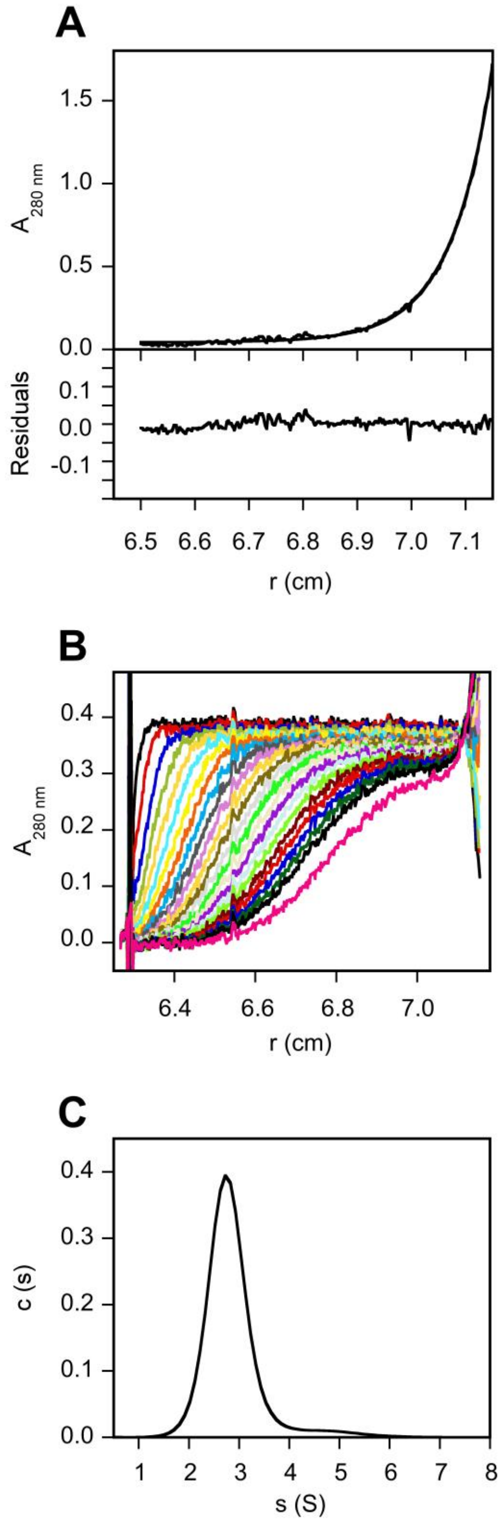

Fig. S3. Analytical ultracentrifugation of *CIRV* p19. (A) Equilibrium run at 18000 rpm, 20°C, (B) sedimentation velocity at 40000 rpm, 20°C, (C) Calculation of the sedimentation coefficient. Analysis of data was performed as described in the Materials and Methods

section. The calculated sedimentation coefficient was s(app) = 2.74 S. From the equilibrium, the corresponding molecular mass was determined to be 46 +/- 4 kDa independently of protein concentration (theoretical mass of *CIRV* p19 monomer 19.479 kDa). These data confirmed the purified p19 to form a protein dimer (22, 23).
