# Supplementary material for: A Viral Suppressor Modulates the Plant Immune Response Early in Infection by Regulating MicroRNA Activity
Source: mBio. 2018 Apr 24;9(2):e00419-18. doi: 10.1128/mBio.00419-18 (PMC5915741; doi:10.1128/mBio.00419-18)
Supplement: FIG S4 [file mbo002183848sf4.pdf]

Fig. S4

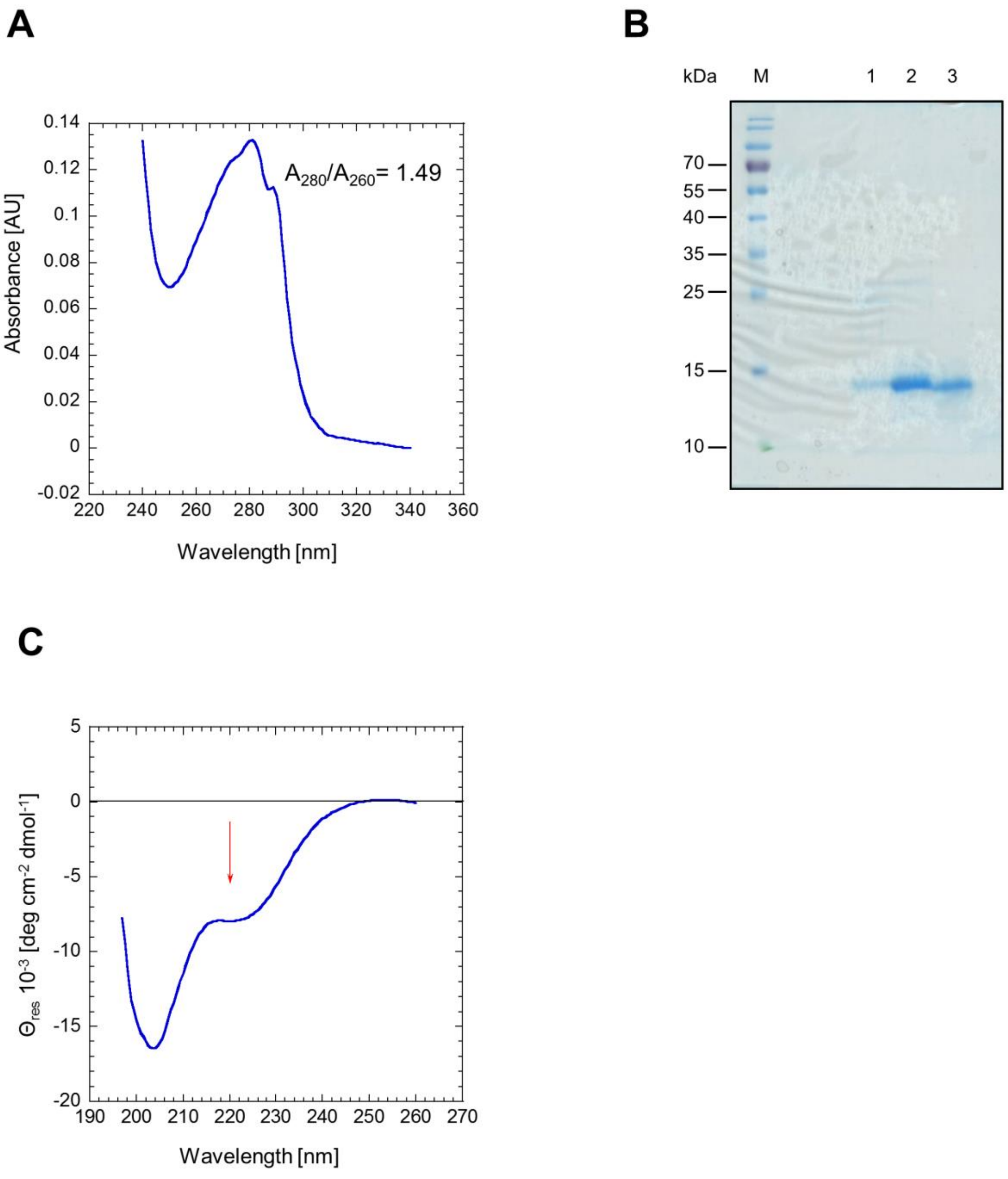

Fig. S4. Purification of *TAV* 2b. (A) Representative absorption spectrum of purified *TAV* 2b. (B) Purified *TAV* 2b gel filtration fractions of ca. 0.1, 0.5 and 0.2 µg of protein were separated on SDS PAGE and stained with coomassie blue. The protein bands run at an apparent size of about 12-13 kDa; mass spectrometry (MALDI TOF) revealed a molecular weight of 11.164 kDa (not shown). (C) Far-UV circular dichroism spectrum (CD)

measurements of the same preparation as in (A) indicate a random coil structure and a small helical content (indicated by the arrow) of the RNA-free protein.
