# Supplementary material for: A Viral Suppressor Modulates the Plant Immune Response Early in Infection by Regulating MicroRNA Activity
Source: mBio. 2018 Apr 24;9(2):e00419-18. doi: 10.1128/mBio.00419-18 (PMC5915741; doi:10.1128/mBio.00419-18)
Supplement: FIG S5 [file mbo002183848sf5.pdf]

Fig. S5

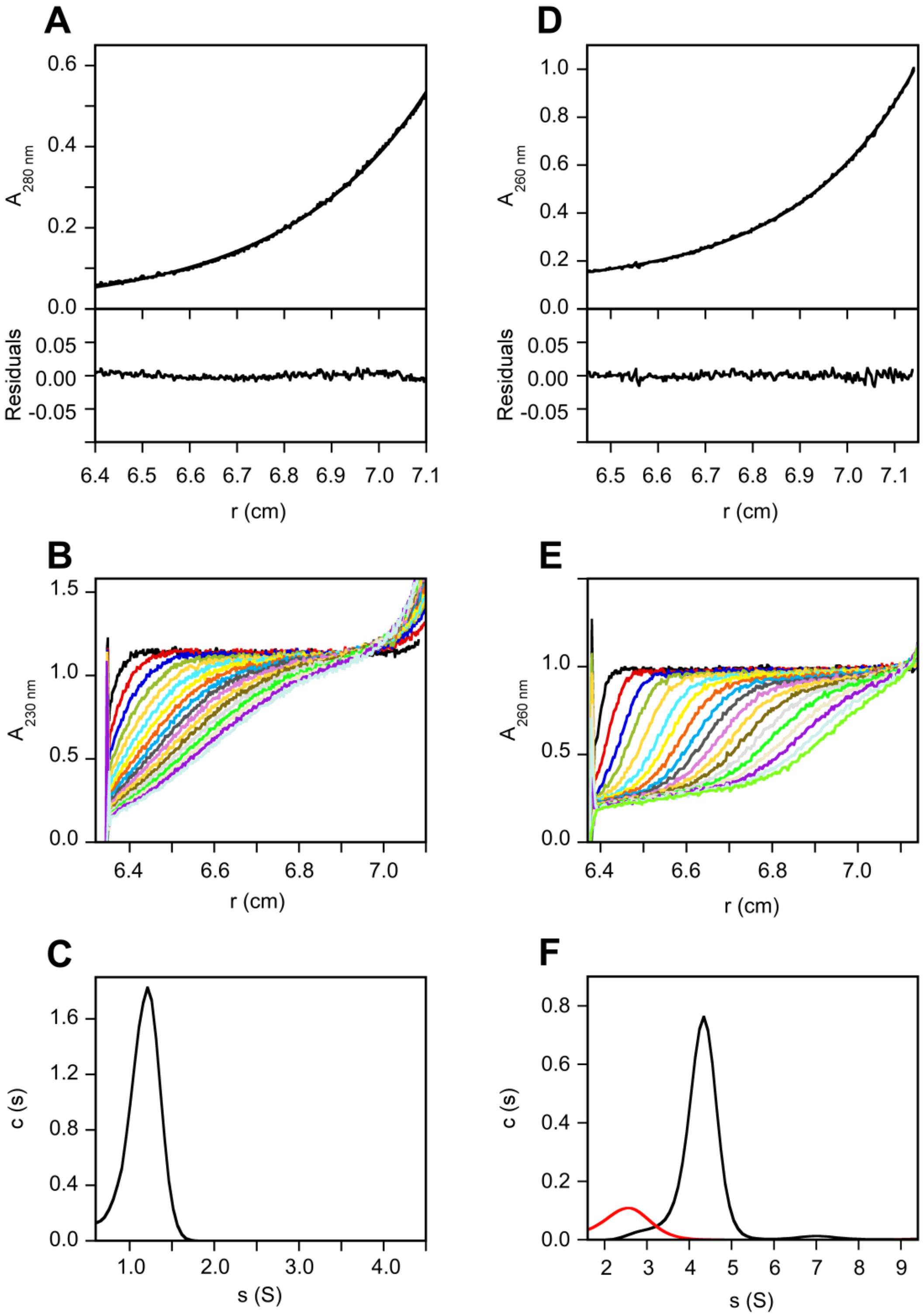


**Fig. S5. Analytical ultracentrifugation of *TAV* 2b in the absence and the presence of miR162.** (A to C) Analyses in the absence of RNA; (D to F) analyses in the presence of miR162. Isolated protein was used at 2 µM, in complex with miR162 the concentrations were

4 µM *TAV* 2b and 2 µM miR162. Data monitoring occurred at 230, 260 or 280 nm. (A) Sedimentation equilibrium of *TAV* 2b (20,000 rpm) yielded an apparent molecular mass of Mr(app) = 10.5 kDa. (B) For sedimentation velocity the sample was measured every 10 min at 40,000 rpm. (C) Data analyses using the program Sedfit resulted in a sedimentation coefficient of s(app) = 1.21 S.(D) Molecular mass determination of the complex was done at 8,000 rpm and yielded a Mr(app) = 51.1 kDa. (E) Sedimentation velocity at 40,000 rpm showed an almost homogenous protein/RNA complex with a sedimentation coefficient of s(app) = 4.34 S (F, black line) together with a small excess of free microRNA (s(app) = 2.55 S (red line in (F)).
