# Supplementary material for: A Viral Suppressor Modulates the Plant Immune Response Early in Infection by Regulating MicroRNA Activity
Source: mBio. 2018 Apr 24;9(2):e00419-18. doi: 10.1128/mBio.00419-18 (PMC5915741; doi:10.1128/mBio.00419-18)
Supplement: FIG S6 [file mbo002183848sf6.pdf]

Fig. S6

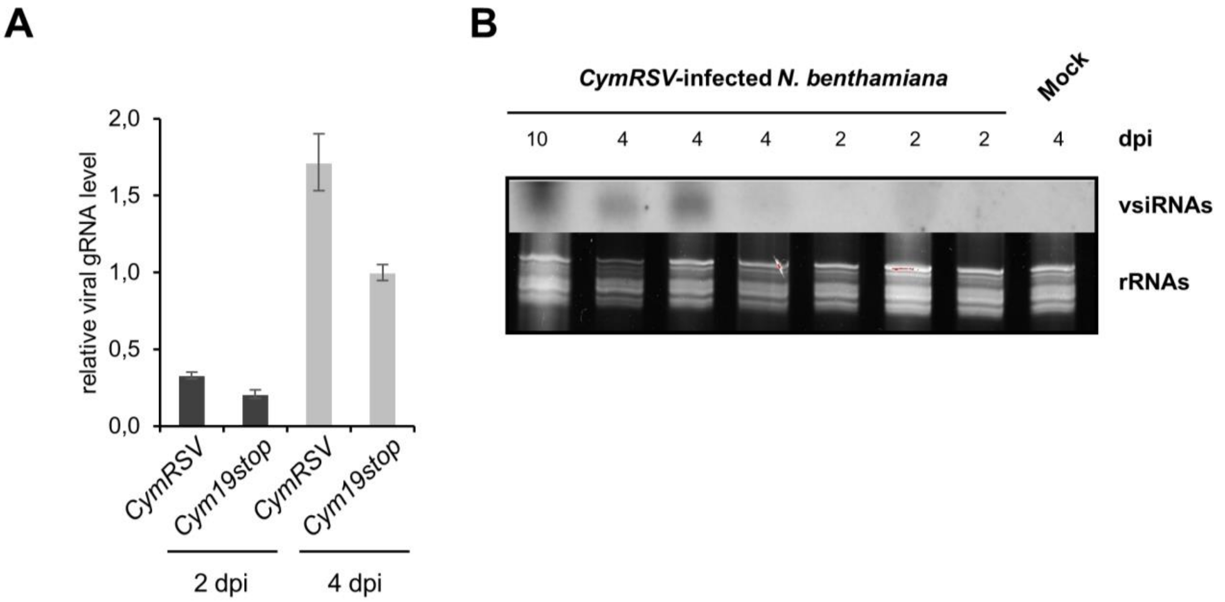


**Fig. S6. Relative accumulation of viral gRNA und virus derived siRNAs in virus-infected *Nicotiana benthamiana* plants.** (A) Relative accumulation of *CymRSV* or *Cym19stop* genomic RNAs as measured by RT-PCR in inoculated leaves of *Nb* at 2 dpi (dark grey bars) and 4 dpi (pale grey bars). All viral gRNA levels differ significantly ($p < 0.05$) between *CymRSV* and *Cym19stop* infected plants at the same day. At 2 dpi, the *Cym19stop* gRNA levels were 63% of that of *CymRSV*. At 4 dpi, the *Cym19stop* gRNA levels were 58% of that of *CymRSV.* (B) Northern blot analysis of *CymRSV* siRNA accumulation in inoculated leaves of *Nicotiana benthamiana* infected plants at 2 and 4 dpi. As a control served one mock-inoculated plant and one *CymRSV*-infected plant at 10 dpi.
