# Supplementary material for: A Viral Suppressor Modulates the Plant Immune Response Early in Infection by Regulating MicroRNA Activity
Source: mBio. 2018 Apr 24;9(2):e00419-18. doi: 10.1128/mBio.00419-18 (PMC5915741; doi:10.1128/mBio.00419-18)
Supplement: FIG S7 [file mbo002183848sf7.pdf]

**Fig. S7**

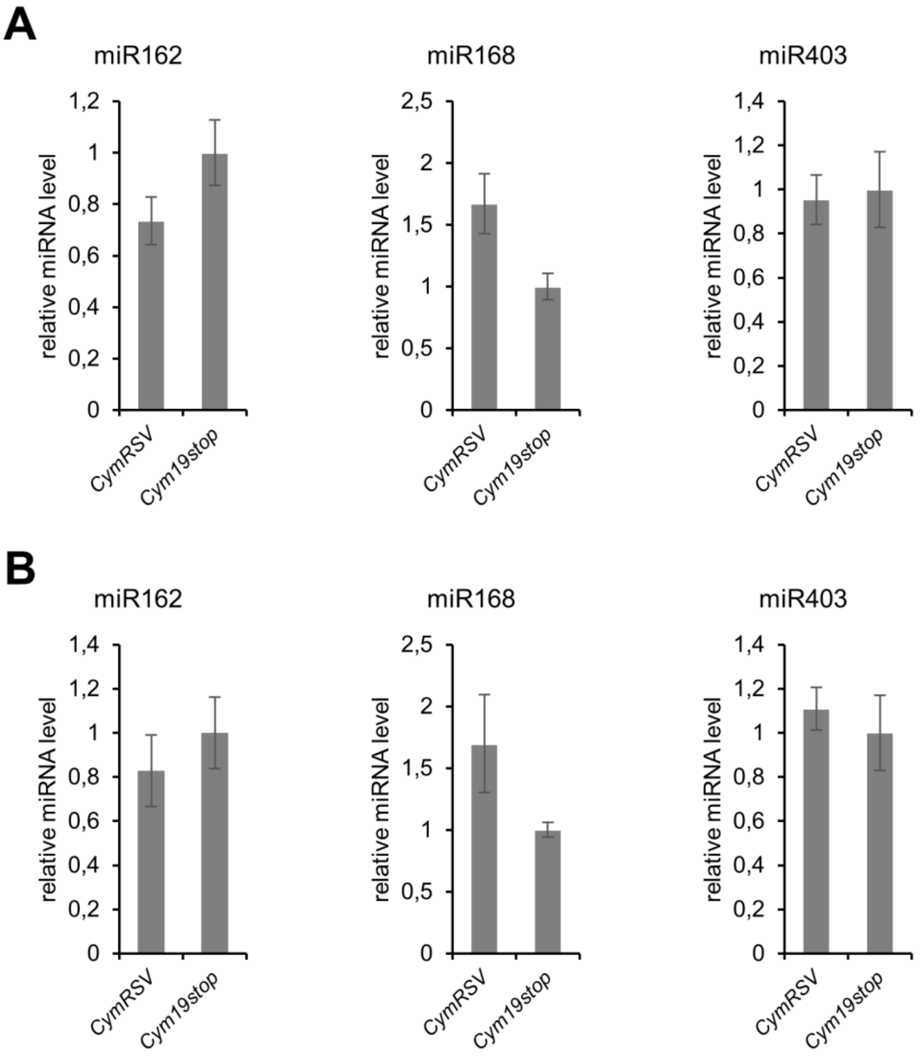


**Fig. S7. Relative accumulation of miRNA guide strands in virus-infected *Nicotiana benthamiana* plants.** Shown are the relative miRNA levels of miR162, miR168 and miR403 guide strands at 2 dpi (A) and 4 dpi (B). Experiments were done in three repetitions. Note that the levels of miR168 were detectably higher in leaves infected with the p19-expressing virus (*CymRSV*) in comparison to *Cym19stop* infected leaves. This was not the case with miR162 and miR403.
