# Supplementary material for: A Viral Suppressor Modulates the Plant Immune Response Early in Infection by Regulating MicroRNA Activity
Source: mBio. 2018 Apr 24;9(2):e00419-18. doi: 10.1128/mBio.00419-18 (PMC5915741; doi:10.1128/mBio.00419-18)
Supplement: FIG S8 [file mbo002183848sf8.pdf]

Fig. S8

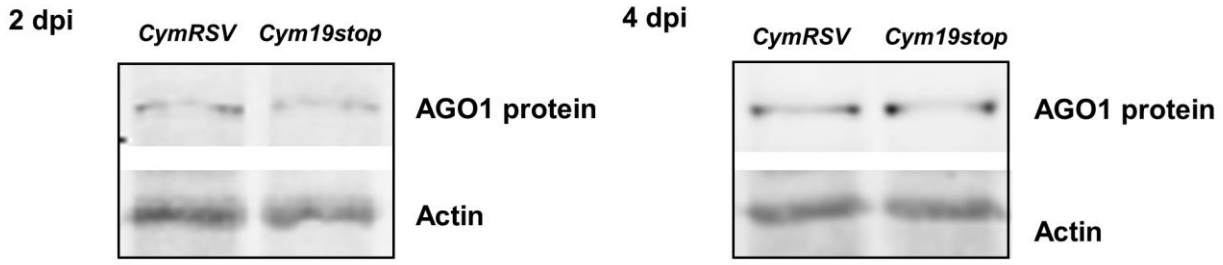

Fig. S8. Western blot analysis of total protein extracts for AGO1 accumulation. *Cym19stop* and *CymRSV* inoculated leaves of *Nicotiana benthamiana* were homogenized at 2 and 4 dpi and used for protein extraction. *Nb*AGO1 protein was detected using a rabbit antibody raised against two N-terminal peptide sequences of the protein. Actin was detected as a loading control. Note that the *Nb*AGO1 levels do not differ between *CymRSV*- and *Cym19stop*-infected plants.
