# Supplementary material for: A Viral Suppressor Modulates the Plant Immune Response Early in Infection by Regulating MicroRNA Activity
Source: mBio. 2018 Apr 24;9(2):e00419-18. doi: 10.1128/mBio.00419-18 (PMC5915741; doi:10.1128/mBio.00419-18)
Supplement: TABLE S1 [file mbo002183848st1.pdf]

Table S1: DNA oligonucleotides used in this study

primer name	sequence	purpose
p19pGEXf	GCGGATCCATGGAACGAGCTA TACAAGGAAAC	PCR primer for p19 cloning (forward)
p19pGEXr	TCCCCCGGGGAATTACTCGCT TTCTTTCTTGAAGGTTTC	PCR primer for p19 cloning (reverse)
pSUMOTAV2b1f	GACCATGAAGACCGTGGTATG GCAAGCATCGAGATCCCTC	PCR primer for *TAV* 2b cloning (forward)
pSUMOTAV2b1r	CCGCTCGAGTTATTGATCGAG ACAC	PCR primer for *TAV* 2b cloning (reverse)
poly(T) adapter	GCGAGCACAGAATTAATACGA CTCACTATAGGTTTTTTTTTTTT VN	reverse transcription
universal reverse primer	GCGAGCACAGAATTAATACGA C	qRT-PCR
5.8S	CCTGCCTGGGCGTCAC	qRT-PCR
U6	GCACAAATCGAGAAATGGT	qRT-PCR
miR403	TTAGATTCACGCACAAACTCG	qRT-PCR of miRNA
miR403*	CGTTTGTGCGTGATTCTGACA	qRT-PCR of miRNA
miR162	CGATAAACCTCTGCATCCAG	qRT-PCR of miRNA
miR162*	AGGCAGCGGTTTATCGATC	qRT-PCR of miRNA
miR168	CTTGGTGCAGGTCGGGAA	qRT-PCR of miRNA
miR168*	CGCCTTGCATCAACTGAAT	qRT-PCR of miRNA
Dcl1f	TTAACTGATGTGTCAGAGTTTG CAAT	qRT-PCR of *DCL1* mRNA
Dcl1r	CAGTCCGAGCAATAAATAAAT CCAT	qRT-PCR of *DCL1* mRNA
Ago1f	TCCTCGGCCACCAGTACC	qRT-PCR of *AGO1* mRNA
Ago1r	TGGCTGAGACGAGGAACCA	qRT-PCR of *AGO1* mRNA
Ago2f	AACCTCCTTTCTATCGACCTAG CA	qRT-PCR of *AGO2* mRNA
Ago2r	GAATGGCAGTAAAACAAACAT GAAAT	qRT-PCR of *AGO2* mRNA
Ago1-1Hf	GCCATGGGGCACCTTCTG	qRT-PCR of *AGO1-1H* mRNA
Ago1-1Hr	GAGACGAGGAACCAGCCTC	qRT-PCR of *AGO1-1H* mRNA
Ago1-1Lf	ATCAACGAGGTGGAGGACAA	qRT-PCR of *AGO1-1L* mRNA

Ago1-1Lr	GGTACTGGTGGCCGTGC	qRT-PCR of *AGO1-1L* mRNA
CPHf	CCAACGGTTCTCAGTTCTTCA	qRT-PCR of *CPH* mRNA
CPHr	CCATACAGCCTCAGCCTTCT	qRT-PCR of *CPH* mRNA
CymRSVf	AGAAATCCTCCAGGACACCTC	qRT-PCR of viral genomic RNA
CymRSVr	AATCCTCTACGCAAAGCAACA GT	qRT-PCR of viral genomic RNA
T7DCL1At1f	CCCTAATACGACTCACTATAG GGTGCATTTACCGATATGC	amplification of miR162 target for *in vitro* slicer assay
TraDCL1At2r	CATCCAACACGATACTCATTAA CC	amplification of miR162 target for *in vitro* slicer assay
T7Ago1Nb2f	CCTAATACGACTCACTATAGG GCACCTCAGCATGGAG	amplification of miR168 target for *in vitro* slicer assay
TraAgo1Nb4r	GCAGATCTTTGTCAGGTAACT C	amplification of miR168 target for *in vitro* slicer assay
T7Ago2Nb1f	CCCTAATACGACTCACTATAG GATCGCAGGTACCCCATACTG	amplification of miR403 target for *in vitro* slicer assay
TraAgo2Nb2r	AAGAGAGAAAATACAAATCAC ATTG	amplification of miR403 target for *in vitro* slicer assay
