# Supplementary material for: A Viral Suppressor Modulates the Plant Immune Response Early in Infection by Regulating MicroRNA Activity
Source: mBio. 2018 Apr 24;9(2):e00419-18. doi: 10.1128/mBio.00419-18 (PMC5915741; doi:10.1128/mBio.00419-18)
Supplement: TABLE S2 [file mbo002183848st2.pdf]

Table S2: RNA oligonucleotides used in this study

oligo name	nucleotide sequence	purpose
gf698 gs	uaguucauccaugccaugugu	guide strand of gf698 siRNA
gf698 ps	acauggcauggaugaacuaua	passenger strand of gf698 siRNA
*At*miR162+	ucgauaaaccucugcauccag	guide strand of *At*miR162 (isoforms a and b) and *Nb*miR162 (isoform a)
*At*miR162*	ggaggcagcgguucaucgauc	passenger strand of *At*miR162 (isoforms a and b) and *Nb*miR162 (isoform a)
*Nb*miR162b*	ggaggcagcgguuuaucgauc	passenger strand of *Nb*miR162 (isoform b)
*At*miR168+	ucgcuuggugcaggucgggaa	guide strand of *At*miR168 (isoforms a and b) and *Nb*miR168 (isoforms d and e)
*At*miR168a*	cccgccuugcaucaacugaau	passenger strand of *At*miR168 (isoform a) and *Nb*miR168 (all isoforms)
*At*miR168b*	cccgucuuguaucaacugaau	passenger strand of *At*miR168 (isoform b)
*Nb*miR168a,b,c+	ucgcuuggugcaggucgggac	guide strand of *Nb*miR168 (isoforms a,b and c)
*At*miR403+	uuagauucacgcacaaacucg	guide strand of *At*miR403 and *Nb*miR403 (both isoforms)
*At*miR403*	uguuuugugcuugaaucuaauu	passenger strand of *At*miR403
*Nb*miR403a*	uguuugugcgugaaucugaca	passenger strand of *Nb*miR403 (isoform a)
*Nb*miR403b*	uguuugugcgugauucugaca	passenger strand of *Nb*miR403 (isoform b)
